# Breaking Kasha’s Rule to Enable Higher Reactivity in Photoredox Catalysis

**DOI:** 10.1021/jacs.5c06115

**Published:** 2025-07-17

**Authors:** Björn Pfund, Oliver S. Wenger

**Affiliations:** Department of Chemistry, 27209 University of Basel, St. Johanns-Ring 19, 4056 Basel, Switzerland

## Abstract

Nearly all photochemical transformations known to date follow Kasha’s rule, implying that reactions occur only from the lowest electronically excited state of a given spin multiplicity due to the fast relaxation of higher-energy states. We challenge this foundational principle by demonstrating with time-resolved laser spectroscopy that the 4,4″-dicyano-*p*-terphenyl radical anion can undergo photoinduced electron transfer directly from a higher-energy excited state, enabling reactivity inaccessible to the lowest excited state with the same spin multiplicity. Preassociation with the substrate and driving-force optimization are critical for overcoming the kinetic barrier to subnanosecond electron transfer, enabling bimolecular anti-Kasha reactivity. This advance establishes a general and broadly applicable framework for bypassing one of the most fundamental principles of photophysics and photochemistry, Kasha’s rule, and opens new possibilities in photoredox catalysis and solar energy conversion by rethinking the energetic and kinetic landscape.

## Graphical abstract

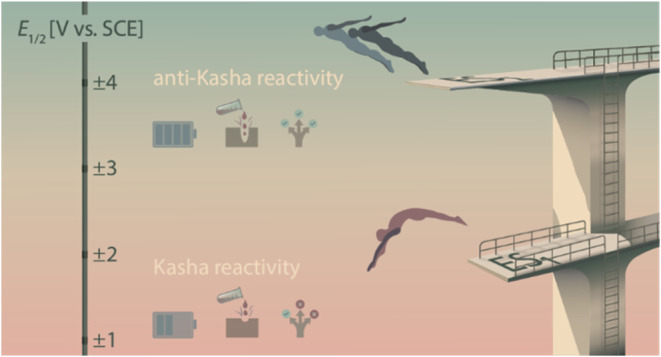

## Introduction

One of the most fundamental principles in photophysics is that light emission usually occurs exclusively from the lowest electronically excited state of a given spin multiplicity, a behavior known as Kasha’s rule.^1^ Higher excited states typically undergo internal conversion to the lowest excited state on a (sub)­picosecond time scale, a rate too fast to allow radiative decay from these higher excited states.^2^ By analogy, photochemical reactions are generally observed to proceed from the lowest excited state of a given spin multiplicity.^3–5^ The ultrafast relaxation of higher excited states dissipates energy that is no longer available for photochemical reactions, limiting the achievable reactivity.^6^ Reactions that would proceed directly from higher excited states, referred as anti-Kasha reactivity, offer an opportunity to unlock new photochemistry, requiring more energy input and higher electrochemical potentials.^7–10^ Several applications could greatly benefit from predictable and reliable access to high-energy (anti-Kasha) photochemistry, including the photodegradation of per- and polyfluoroalkyl substances (PFAS), the conversion of sunlight to chemical fuels, and synthetic photochemistry targeting fine chemicals.

Any photochemical activation step of substrate molecules must effectively compete with the relaxation processes on a photocatalyst to enable productive chemical reactions.^11,12^ Most photocatalytic systems rely on bimolecular diffusion to bring the substrate (Sub) to react with the excited photocatalyst (*PC), yielding a maximum reaction rate (*k*_R_) of approximately 10^9^ s^–1^, which is the diffusion-limited rate at typical substrate concentrations (Figure 1a).^5^ This requires natural excited-state lifetimes (τ_0_) in the nanosecond regime or longer to effectively compete against intrinsic decay processes.^13^ Consequently, under such diffusion-based conditions, bimolecular reactivity can usually only originate from the lowest excited state of a given spin multiplicity, as internal conversion rates (*k*_IC_) from the second excited states (ES_2_) to the lowest excited state (ES_1_) are orders of magnitude faster than the diffusion-limited reaction rate. Diffusion-based photochemistry, therefore, commonly follows Kasha’s rule, making the available redox potentials independent of the excitation energy (Figure 1c, orange decay pathway).

**Figure 1:**
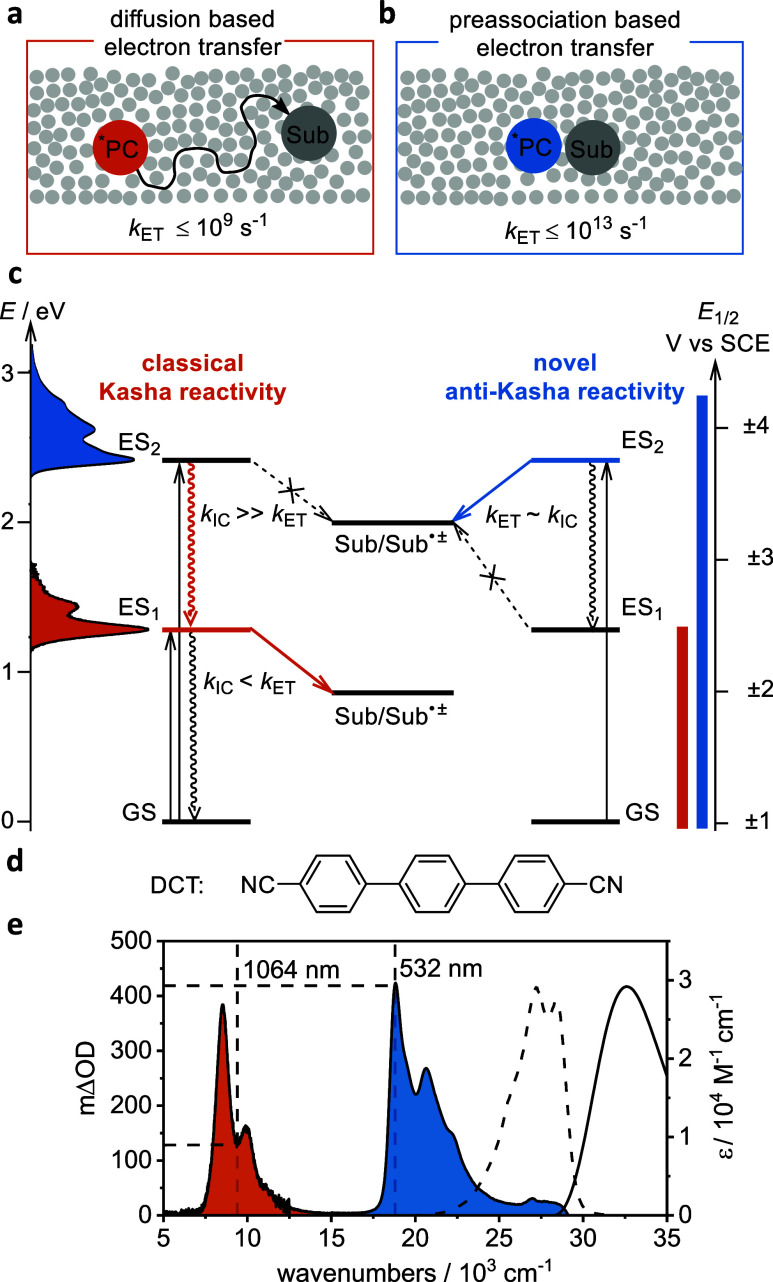
(a) Classical photoredox reactions rely on bimolecular diffusion for substrate (Sub) activation by the excited photocatalyst (*PC), limiting the electron transfer rate (*k*_ET_) to ≤10^9^ s^–1^ at typical substrate concentrations, requiring at least nanosecond-scale excited state lifetimes. (b) Preassociation between Sub and *PC enables electron transfer rates of up to 10^13^ s^–1^, facilitating reactions of much shorter-lived excited states. (c) Simplified energy diagram of classical Kasha reactivity (left), where internal conversion rates (*k*_IC_) of up to ∼10^13^ s^–1^ to the lowest excited state (ES_1_) prevent reactivity from higher excited states (ES_2_), causing significant energy loss. Anti-Kasha reactivity (right): preassociation between PC and Sub enables competitive *k*_ET_, allowing ultrafast electron transfer from higher excited states to form redox products (Sub^•±^) inaccessible from ES_1_. (d) Molecular structure of 4,4″-dicyano-*p*-terphenyl (DCT). (e) UV–vis absorption spectrum (solid black line), and normalized luminescence spectrum (dashed black line) of DCT in DMF. Absorption changes following the one-electron reduction of DCT in DMF are shown in orange and blue, representing the absorption bands resulting from excitation into the D_1_ ([^2*^DCT^•–^]_D_1__) and D_2_ ([^2*^DCT^•–^]_D_2__) excited states, respectively. The dashed straight lines indicate the excitation wavelengths of the two-color pump–pump–probe experiments.

Few compounds, like azulenes and zinc­(II) porphyrins, exhibit much slower internal conversion rates due to an unusually large energy gap between ES_1_ and ES_2_, allowing competitive radiative decay of ES_2_ with luminescence lifetimes of around 1 ps up to a few nanoseconds.^14–70^ These excited state lifetimes enable anti-Kasha reactivity when zinc­(II) porphyrins are covalently linked or preassociated with a suitable reaction partner.^16–24^ This preassociation is essential for enabling static photoinduced electron transfer (ET) with reaction rates reaching up to 10^13^ s^–1^ (Figure 1b).^25,26^ Consequently, static quenching^27,28^ offers potential for systematic access to anti-Kasha reactivity, which is of interest, among other areas, in dye-sensitized solar cells (DSSCs) and water-splitting dye systems, where higher excited states can inject an electron into the covalently bound semiconductor.^29,30^ However, solution based bimolecular anti-Kasha reactivity remains largely unexplored, and is generally considered unattainable for most photocatalysts owing to their ultrafast internal conversion rates requiring femtosecond reactivity—a condition viewed as unrealistic even with preassociation.^5^ While some synthetic studies have speculated about the possibility of anti-Kasha reactivity in excited organic radicals as photocatalysts^31,32^ and kinetic analysis suggests the possibility of femtosecond reactivity,^5^ spectroscopic evidence has been absent.

In this study, we observe anti-Kasha reactivity from 4,4″-dicyano-*p*-terphenyl (Figure 1d) radical anion (DCT^•–^), a strong photoreductant. By leveraging preassociation between DCT^•–^ and substrate molecules^25^ we achieve static ET rates that compete against nonproductive internal conversion. Using two-color pump–pump–probe laser flash photolysis we directly observe ultrafast ET from higher-energy excited states. Our results reveal a threshold ET driving force required to achieve anti-Kasha reactivity, establishing design principles for photocatalytic systems capable of exploiting this phenomenon. This work lays the foundation for broad applications of anti-Kasha reactivity and redefines the energetic landscape of photocatalysis.^33^

## Results and Discussion

### Direct Observation of Anti-Kasha Reactivity

Based on the UV–vis absorption spectrum of ^2^DCT^•–^ (Figure 1e), the electronic transitions from the doublet ground state (D_0_) to the first (D_1_) and second (D_2_) doublet excited states yield [^2*^DCT^•–^]_D_1__ and [^2*^DCT^•–^]_D_2__ energies (*E*_D_) of 1.0 and 2.3 eV, respectively. Using the one-electron reduction potential (*E*_red_) of DCT at −1.7 V versus SCE (Figure S1), the reduction potentials of the D_1_ and D_2_ excited states (^2*^ *E*_red_) are estimated using the Rehm–Weller equation,^34^ as approximately −2.7 and −4.0 V versus SCE (Figure S2). While the Rehm–Weller formalism is established for excited organic radicals,^35,36^ its applicability to higher excited states is not yet established, though it appears plausible to us. Nonetheless, the 1.3 V potential difference between D_1_ and D_2_ likely leads to distinct reactivity profiles.

Ultrafast transient absorption spectroscopy initially seems to be the most logical approach to investigate the proposed subpicosecond anti-Kasha reactivity.^36^ However, in our system, the anticipated static quenching between [^2*^DCT^•–^]_D_2__ and the electron acceptor (Sub) would further reduce the already weak transient absorption signal, as the potentially ultrafast electron transfer to the substrate may occur within the instrument response function, making detection challenging.^25^ Furthermore, a two-color pump–pump–probe femtosecond transient absorption experiment (or a combination of spectro-electrochemistry and subpicosecond pump–probe spectroscopy)^37,71^ would be required to first generate the active photocatalyst DCT^•–^, further limiting the accessibility of such measurements.^38^ To overcome this technical limitation, we employed nanosecond two-color pump–pump–probe spectroscopy (Figure 2a).^25,39^ In this experiment, direct excitation of DCT with 355 nm laser pulses in the presence of an electron donor (D_sac_), such as *N*,*N*-dimethylaniline (DMA), generates DCT^•–^. After a delay of several microseconds, a second laser pulse at either 1064 or 532 nm selectively excites DCT^•–^, forming [^2*^DCT^•–^]_D_1__ or [^2*^DCT^•–^]_D_2__, respectively. Without an electron acceptor, these excited states decay back to the doublet ground state (DCT^•–^) within the 10 ns duration of the second laser pulse, resulting in no change in the transient absorption dynamics. When an electron acceptor capable of oxidizing the electronically excited DCT^•–^ is present, the second excitation triggers an ET reaction. This results in an instantaneous bleach of the DCT^•–^ transient absorbance signal, as ET from ^2*^DCT^•–^ to the acceptor forms charge-neutral DCT and a (neutral) phenyl radical along with halogenide anions, which do not absorb in the visible region.^25,40^

**Figure 2:**
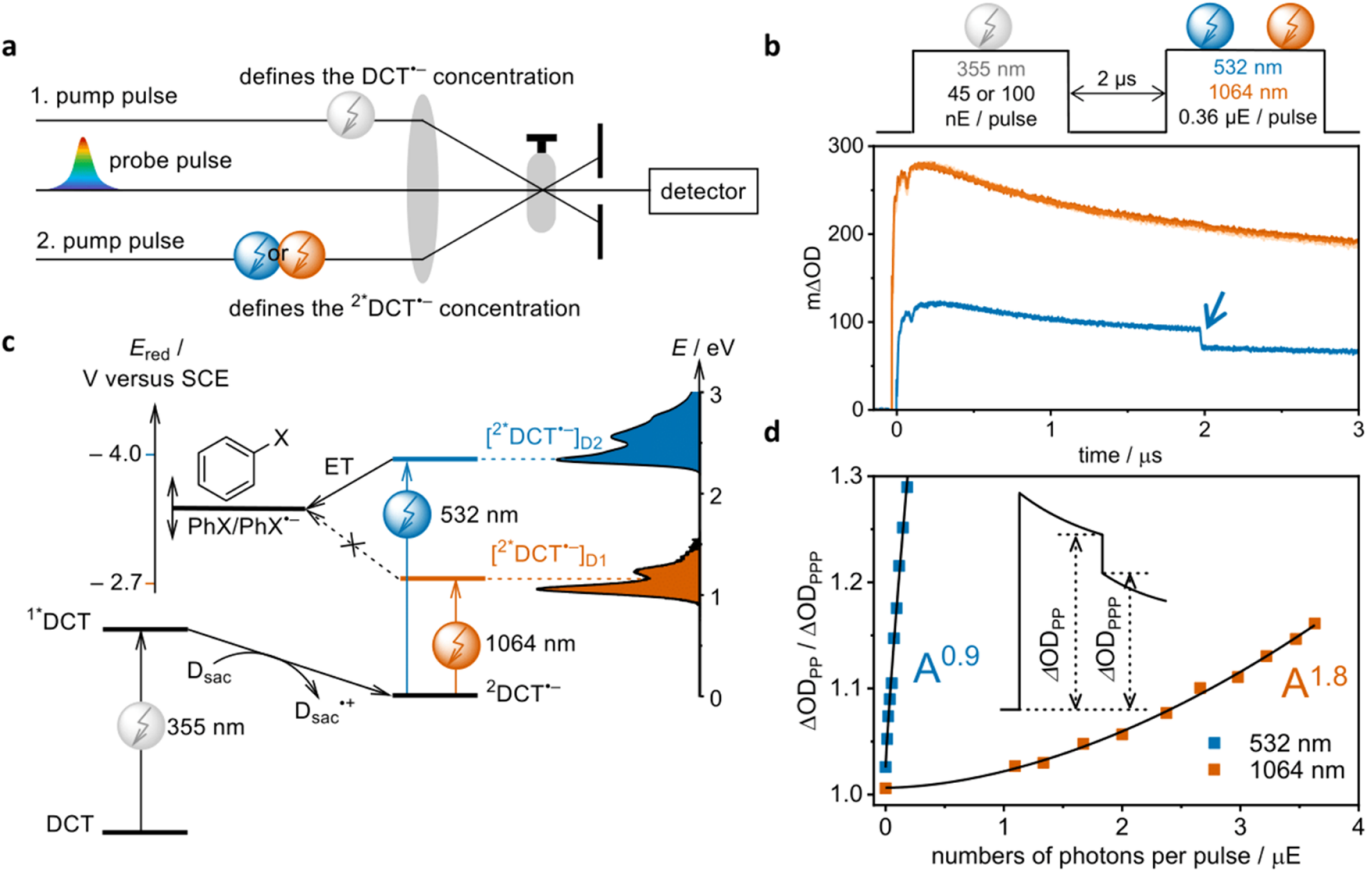
(a) Schematic representation of the two-color pump–pump–probe experiment. (b) Transient absorption kinetics for DCT^•–^ detected at 500 nm with 1 M chlorobenzene as an electron acceptor. Electron transfer occurs selectively from the higher excited state populated with 532 nm excitation (blue trace, reaction marked by a blue arrow), whereas the lowest excited state (orange trace) remains unreactive. (c) Simplified energy diagram. Excitation of DCT at 355 nm with a sacrificial electron donor (D_sac_) present generates DCT^•–^. Using 1064 or 532 nm light, DCT^•–^ is selectively excited into the first ([^2*^DCT^•–^]_D_1__) or second ([^2*^DCT^•–^]_D_2__) doublet excited state. In the presence of an electron acceptor with a reduction potential between those of the D_1_ and D_2_ states of DCT^•–^, electron transfer (ET) occurs selectively from [^2*^DCT^•–^]_D_2__. (d) Power dependency of the second laser pulse at 532 nm (blue trace) and 1064 nm (orange trace) using chlorobenzene. (Inset) Illustration of the two key observables: ΔOD_PP_ (PP = pump–probe, change in optical density before the second pump pulse) and ΔOD_PPP_ (PPP = pump–pump–probe, change in optical density after the second pump pulse).

To explore the anticipated anti-Kasha reactivity (Figure 2c), chlorobenzene was initially selected as the electron acceptor, with a reduction potential of −2.8 V versus SCE lying between the redox potentials of [^2*^DCT^•–^]_D_1__ and [^2*^DCT^•–^]_D_2__. Under these conditions, excitation at 1064 nm, which populates the D_1_ state, should not induce ET with chlorobenzene. In contrast, 532 nm excitation populates the D_2_ state, providing sufficient reduction power expected to drive a fast ET reaction, resulting in the characteristic bleach of the transient absorption signal (Figure 2b).

Given that the absorption coefficient of DCT^•–^ at 532 nm is ∼2.5 times higher than at 1064 nm, the 355 nm laser pulse energy was tuned to equalize the transient absorption signal intensities at both wavelengths. Since both transient absorption kinetics were monitored at 500 nm, a ∼2.5 times stronger signal was targeted for the 1064 nm excitation to compensate for the lower absorption at that wavelength. This ensures a valid comparison by maintaining equimolar photon absorbance at 1064 and 532 nm (see Supporting Information). Using a deaerated DMF solution containing DCT, DMA, and 1 M chlorobenzene, direct excitation of DCT with 355 nm pulses generated DCT^•–^. A subsequent 532 nm pulse caused an instantaneous bleach of the DCT^•–^ absorbance, indicating ET from the [^2*^DCT^•–^]_D_2__ state to chlorobenzene, as anticipated. In contrast, excitation at 1064 nm resulted in undetectable changes in the transient absorption signal (Figure 2b, orange trace), consistent with the insufficient reduction potential of the [^2*^DCT^•–^]_D_1__ state for ET with chlorobenzene.

Photoreactivity after D_1_ excitation remained undetectable unless very high pulse energies of up to 410 mJ per pulse were applied, delivering ∼10 times more photons than 532 nm excitation (Figure S3). An excitation power-dependent study was conducted to distinguish between one- and two-photon excitation processes by monitoring the induced bleach of DCT^•–^. The bleach was quantified by comparing the changes in optical densities (ΔOD) at 500 nm, determined immediately before the second pump pulse (ΔOD_PP_; PP = pump–probe) and shortly afterward (ΔOD_PPP_; PPP = pump–pump–probe).^25,39^ To avoid potential optical artifacts, the ΔOD_PPP_ values were extracted by averaging the signal in a time window of 75 ns starting approximately 50 ns after the second laser pulse. A plot of ΔOD_PP_/ΔOD_PPP_ against the number of photons used reveals a linear relationship for 532 nm excitation, consistent with a monophotonic mechanism (Figure 2d, blue trace). In contrast, 1064 nm excitation showed a quadratic dependency, indicating a two-photonic mechanism (Figure 2d, orange trace). This suggests a two-photon absorption of DCT^•–^ or further excitation of [^2*^DCT^•–^]_D_1__, both leading to the population of the [^2*^DCT^•–^]_D_2__ state from which reaction with chlorobenzene is possible. These results indicate that anti-Kasha reactivity is accessible both via conventional monophotonic processes as well as via two-photon excitation events. While it is reasonable to assume that no decomposition product absorbs at 1064 nm, this assumption is less certain at 532 nm, where more organic chromophores typically absorb.^41,42^ To address this, we performed a pseudoexcitation (or “photoaction”) spectrum by keeping the first pulse constant while varying the excitation wavelength of the second laser pulse between 420 and 510 nm. The observed bleach at different excitation wavelengths was compared to the UV–vis absorption spectrum of DCT^•–^, showing good alignment within the accessible wavelength range (Figure S5). This result confirms reactivity originating from the [^2^DCT^•–^]_D_2__ excited state.

### Exploring the Thermodynamic Limits

To explore the thermodynamic and kinetic limits of the anti-Kasha reactivity exhibited by DCT^•–^, we tested fluorobenzene as an electron acceptor, with a reaction free energy of −1.0 eV for photoinduced electron transfer from the D_2_ excited state (Δ*G*_ET(D_2_)_^0^). However, no reaction was observable indicating that photoinduced electron transfer is kinetically outcompeted by internal conversion. This finding most likely reflects a too high activation barrier for ET resulting from an imbalance between driving-force and reorganization energy. To systematically explore the driving-force dependence of potential anti-Kasha reactivity and conventional Kasha reactivity, we conducted two-color pump–pump–probe experiments with electron acceptors having reduction potentials ranging from −3.0 to −1.2 V versus SCE (Figure 3e). While accurately determining the thermodynamic redox potentials of this family of quenchers is challenging due to their irreversible redox behavior, cyclic voltammetry has been shown to provide reasonable estimates.^43^

**Figure 3:**
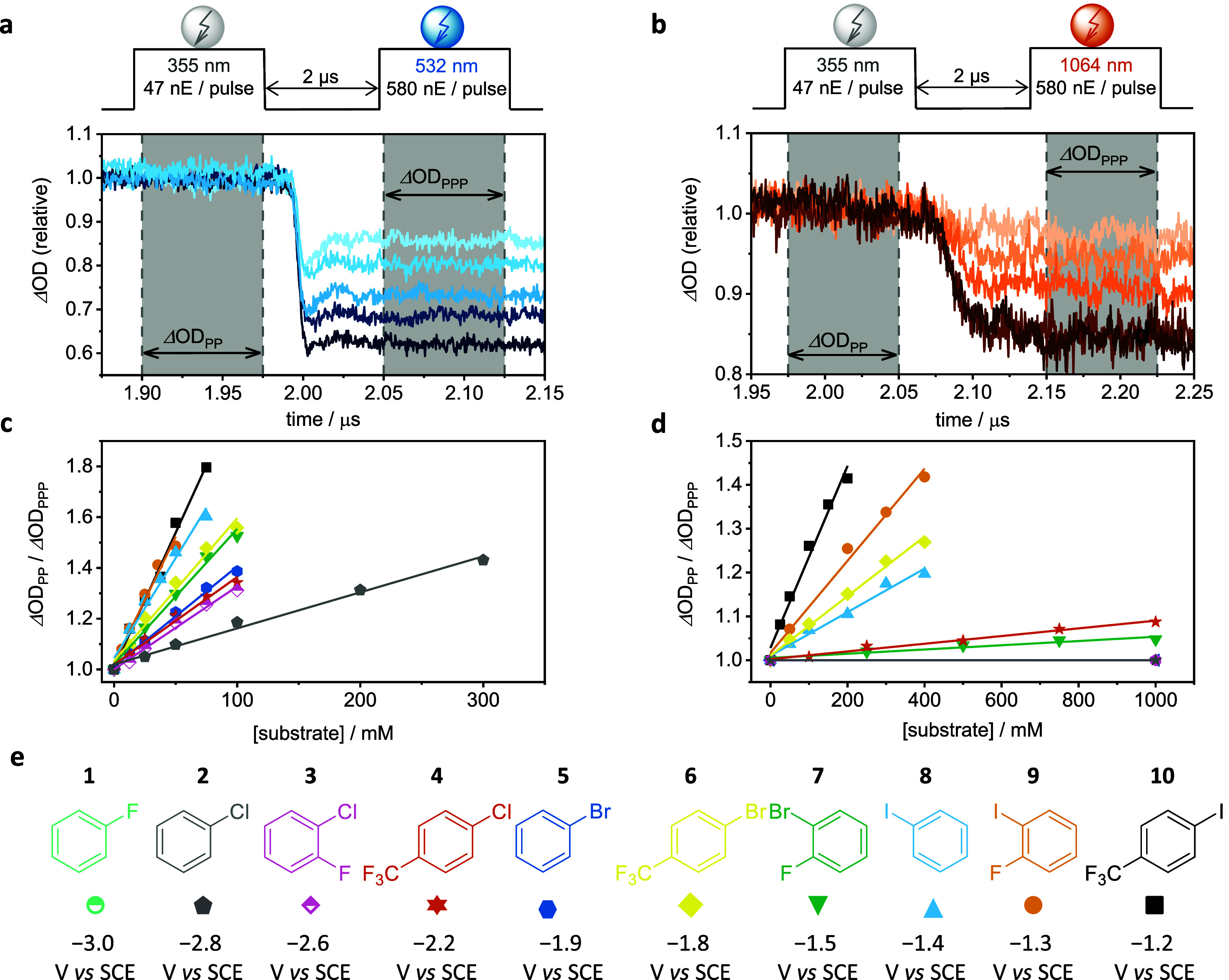
(a, b) Transient UV–vis absorption kinetic decay of DCT^•–^ monitored at 500 nm in a two-color pump–pump–probe experiment, with the pulse scheme illustrated at the top. A 355 nm laser pulse (16 mJ) first generates DCT^•–^. After a delay of 2 μs, a second laser pulse of 532 nm (a) or 1064 nm (b) excites DCT^•–^. The experiment used an argon-saturated DMF solution containing 2 mM DCT, 200 mM DMA, and varying iodobenzene concentrations. The gray shaded areas indicate the range of the averaged signal intensity for ΔOD_PP_ (PP = pump–probe, changes in optical density immediately before the second pump pulse) and ΔOD_PPP_ (PPP = pump–pump–probe, change in optical density recorded after the second pump pulse). To avoid potential optical artifacts, the ΔOD_PPP_ values were extracted by averaging the signal over a 75 ns time window starting approximately 50 ns after the second laser pulse. (c, d), Stern–Volmer type plot based on the two-color pump–pump–probe experiments performed at varying electron acceptor concentrations. (e) Chemical structures of the electron acceptors used, with their reduction potential against saturated calomel electrode (SCE). Color- and symbol-codes correspond with panels (c, d).

Specifically, we compared the signal bleach induced by a pulse at 532 nm, exciting the D_2_ excited state, and 1064 nm, exciting the D_1_ excited state, at different electron acceptor concentrations. Exemplary experiments with iodobenzene as the electron acceptor are shown in Figure 3a,b, while results with other acceptors are presented in Figures S6–S20. Titration experiments with electron acceptors at 12.5 to 1000 mM concentrations revealed progressively larger bleaches. The effect of the 532 or 1064 nm pulse was quantified by comparing the changes in optical densities (ΔOD) at 500 nm, measured immediately before the pulse (ΔOD_PP_, PP = pump–probe) and shortly afterward (ΔOD_PPP_, PPP = pump–pump–probe). These values were determined by averaging signal intensities within the gray-shaded areas of Figure 3a,b. To avoid potential optical artifacts, the ΔOD_PPP_ values were extracted by averaging the signal over a 75 ns time window starting approximately 50 ns after the second laser pulse. A pseudo Stern–Volmer analysis was performed by plotting ΔOD_PP_/ΔOD_PPP_ against the electron acceptor concentration (Figure 3c,d).^39^

As the electron acceptor concentration increased, a linear Stern–Volmer relationship was observed for both excitation wavelengths, indicating a first-order reaction with respect to the electron acceptors (Table S1). For 532 nm excitation, the pseudo Stern–Volmer constant (*K*_SV_) showed a direct correlation with the increasing driving force (Figure 4a). For 1064 nm excitation (populating the [^2*^DCT^•–^]_D1_ state), overall significantly smaller *K*_SV_ values were obtained (Figure 4b). For reaction free energies (Δ*G*_ET(D_1_)_^0^) less negative than −0.9 eV, the *K*_SV_ values were too small to be determined in most cases (Table S1). Evidently, far more effective photoreactivity is observed after excitation of DCT^•–^ into its D_2_ excited state compared to D_1_ excitation, illustrating the power of anti-Kasha reactivity.

**Figure 4:**
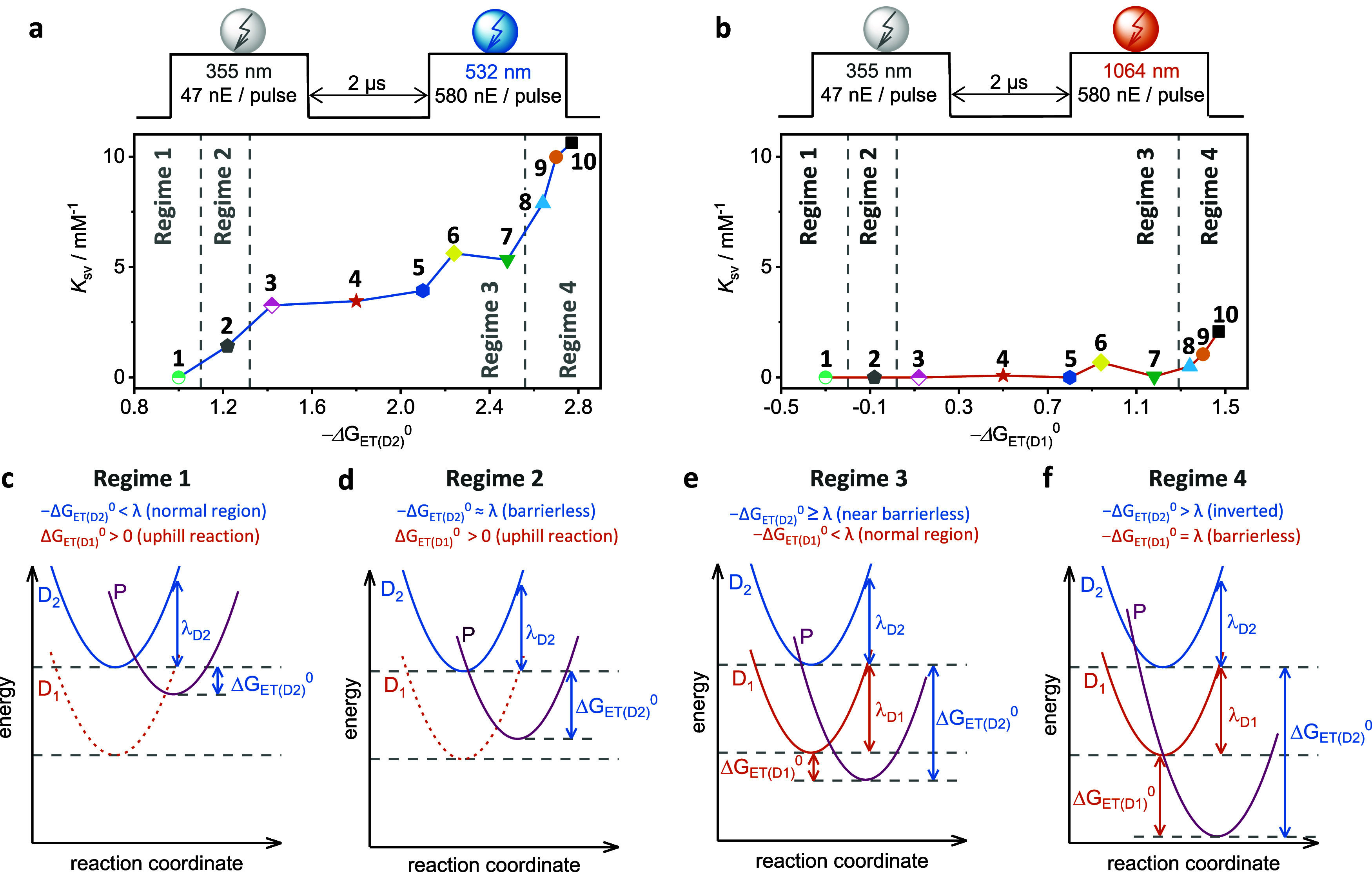
(a) Stern–Volmer constants (*K*_SV_) obtained for D_2_ excitation using 532 nm are plotted against the estimated driving force for photoinduced electron transfer from the D_2_ excited state to the different acceptors (Figure 3e), with the pulse scheme illustrated above. (b) The *K*_SV_ values obtained for direct D_1_ excitation using 1064 nm are plotted against the estimated driving force for photoinduced electron transfer from the D_1_ state to the different acceptors (Figure 3e) with the pulse scheme illustrated above. (c–f) The potential energy well diagrams illustrate four different kinetic regimes for photoinduced electron transfer (ET) between [^2^DCT^•–^]_D_2__ (blue parabola) or [^2^DCT^•–^]_D_1__ (orange parabola) and the electron acceptors from Figure 3e to form photoproducts P (= DCT and reduced acceptor, purple parabola). The key point is to achieve barrierless electron transfer, by increasing the amount of the reaction free energies (Δ*G*_ET(D_2_)_^0^ or (Δ*G*_ET(D_1_)_^0^) to the point that they equal the reorganization energy (λ). This point is reached in (d) for the D_2_ excited state and in (f) for the D_1_ excited state of DCT^•–^.

### Decoding Anti-Kasha Behavior via Marcus Theory

The data in Figure 4a,b suggest a complex interplay between the driving force and the contributions of the D_1_ and D_2_ excited states to the overall reactivity. The energy-level diagram in Figure 2c cannot adequately explain this interplay. Instead, we use Marcus’ theory for electron transfer to qualitatively distinguish between four different regimes that account for the combined D_1_ and D_2_ photoreactivity data.

The first regime (Figure 4c) describes the situation for the weakest electron acceptors, where photoinduced electron transfer is exergonic from the D_2_ excited state but endergonic from the D_1_ state. In this regime, the driving force for electron transfer from the D_2_ excited state (Δ*G*_ET(D_2_)_^0^) is assumed to be smaller than the reorganization energy (λ) leading to a significant activation barrier for electron transfer. This barrier likely accounts for the absence of observable D_2_ reactivity with fluorobenzene (**1**), the weakest acceptor investigated. This barrier persists despite a thermodynamic driving force of 1.0 eV from the D_2_ state of DCT^•–^, making photoinduced electron transfer kinetically uncompetitive with internal conversion (Figure 1c). The lifetime of the D_2_ excited state has not been determined but is likely in the picosecond range or shorter,^44^ corresponding to an internal conversion rate (*k*_IC_) on the order of 10^12^–10^13^ s^–1^. To observe ET from D_2_, the electron transfer rate (*k*_ET_) must compete with *k*_IC_ (Figure 1c). This necessitates *k*_ET_ to be near to its theoretical upper limit, which is expectable when the activation barrier is close to zero.^45^ This condition is met when the reaction free energy (Δ*G*_ET(D_2_)_^0^) equals the total reorganization energy (λ) of the electron transfer reaction.

Overall (inner and outer sphere) reorganization energies are typically between 0.8 and 1.2 eV in molecular systems.^45,46^ Hence, in such a simple picture activation free electron transfer is expected for driving forces in this range. This point is not reached with fluorobenzene (Δ*G*_ET(D_2_)_^0^ ≈ −1.0 eV), where the experiment suggests *k*_IC_ > *k*_ET_ (Figures 4a and 1c). Additionally, reverse electron transfer from the primary photoproducts (purple parabola, Figure 4c) to a lower excited D_1_ state (orange parabola, Figure 4c), may further limit the observable reactivity.^47^ However, it is important to note that these two-color two-pulse experiments require an ET efficiency of 5–10% for detection.^25^ In contrast, under photoredox conditions with continuous light irradiation for hours, much smaller efficiencies can sustain reactivity.^48^

The second regime (Figure 4d) presents a similar scenario, in which photoinduced electron transfer is exergonic from the D_2_ excited state but remains endergonic from the D_1_ state. In contrast to the first regime, the driving force of the D_2_ excited state (Δ*G*_ET(D_2_)_^0^) is now comparable to the reorganization energy (λ), resulting in a nearly barrierless electron transfer. This regime is in line for chlorobenzene (**2**), where reactivity is solely observed from the D_2_ excited state with an estimate driving force of Δ*G*_ET(D_2_)_^0^ ≈ −1.2 eV (Figure 3), which evidently makes the electron transfer rate competitive with the internal conversion rate (Figure 1c). This aligns with previous studies, showing that *k*_ET_ can reach values between 10^12^ and 10^13^ s^–1^ for driving forces above 1.0 eV.^49–51^ Further, reverse charge shift to the D_1_ excited state in this regime (Figure 4d) is likely reduced compared to the first regime (Figure 4c) due to the higher activation barrier for this unwanted process.

The third regime (Figure 4e) represents a scenario where photoinduced electron transfer is thermodynamically accessible from both the D_2_ and the D_1_ excited state. Electron transfer from the D_2_ excited state remains near barrierless or is slightly inverted, while the ET rate from D_1_ remains limited due to a relatively small driving force (Δ*G*_ET(D_1_)_^0^), introducing a significant activation barrier. This behavior is exemplified by the next five electron acceptors (**3**–**7**). Upon D_2_ excitation, fast ET is consistently observed (Figure 4a), compatible with the near-barrierless regime. In contrast, despite a driving force from D_1_ with Δ*G*_ET(D_1_)_^0^ values ranging from −0.1 to −1.2 eV no significant reactivity is observed (Figure 4b). This indicates that for these acceptors *k*_ET_ is significantly smaller compared to *k*_IC_ due to the activation barrier imposed by insufficient driving force (Figure 4e). Given the D_1_ excited-state lifetime of DCT^•–^ is ∼1.1 ps,^25^ the corresponding *k*_IC_ is ∼ 1 × 10^12^ s^–1^, supporting this analysis similarly as discussed above (regime 1) for the D_2_ excited state where no reaction occurs with fluorobenzene (**1**). Photoreactivity from the D_2_ state with acceptors **3**–**7** occurs with pseudo Stern–Volmer constants (*K*_SV_) in a narrow range of 3 to 5 mM^–1^, with no evidence for a Marcus inverted driving-force effect, where *k*_ET_ decreases when the reaction free energy significantly exceeds the reorganization energy.^49^ This inverted effect can weaken due to nuclear tunneling,^46^ and the commonly used harmonic potential-well picture becomes simplistic at high driving-forces. Hence, the relatively constant D_2_ photoreactivity with acceptors **3**–**7** is not necessarily surprising.

The fourth regime (Figure 4f) represents systems with the highest driving forces studied, where photoinduced electron transfer is feasible from both D_2_ and D_1_ excited states. As the driving force increases further, the D_2_ excited state falls more clearly into the Marcus inverted region, which in principle could result in a sizable activation barrier that could slow ET despite the strong thermodynamic driving force, but no deceleration is observed. The less reactive D_1_ excited state now enters the barrierless regime, where ET proceeds very rapidly. This scenario is reached with iodobenzene (**8**), 2-fluoro-1-iodobenzene (**9**), and 4-iodobenzotrifluoride (**10**), for which D_1_ photoreactivity is detectable in the Δ*G*_ET(D_1_)_^0^ range from −1.3 to −1.5 eV (Figure 4b). Thus, the activation barrier becomes sufficiently low for D_1_ reactivity at a Δ*G*_ET(D_1_)_^0^ value of −1.3 eV, similar to the threshold value of Δ*G*_ET(D_2_)_^0^ of −1.2 eV necessary to observe D_2_ reactivity. For acceptors **8**–**10**, the apparent D_2_ reactivity observed after excitation at 532 nm further increases compared to *K*_SV_ values for acceptors **3**–**8** (Figure 4a). This is likely due to additional D_1_ reactivity after internal conversion from D_2_ to D_1_. Thus, reactivity in the fourth regime reflects contributions from both excited states, complicating the isolation of the higher excited states reactivity. For the most readily reducible electron acceptors (**8**–**10**), *K*_SV_ values obtained after D_2_ excitation are about four times higher than those *K*_SV_ values obtained after D_1_ excitation (Figure 4a,b). The electronic coupling between the reactant and product potential wells in Figure 4 may differ depending on the involved D_1_ or D_2_ excited state, and even small differences in electronic coupling could influence the reaction kinetics here. Additionally, unlike classical (luminescence) quenching experiments, our two-color pump–pump–probe experiments monitor electron transfer including successful cage escape, since in-cage charge recombination regenerates the original species prior to the second laser pulse, resulting in no net signal change (Figure S22). As cage escape efficiency is known to be driving force dependent in some cases,^52–54^ the D_2_ state can be expected to exhibit higher reactivity compared to D_1_, potentially explaining the enhanced reactivity observed for excited radicals under higher-energy light illumination.^31,32,55^

### The Future of Anti-Kasha Reactivity

This study demonstrates that productive photoinduced electron transfer in solution can occur directly from a higher-energy excited state of an organic radical, challenging the long-standing principle that photochemical reactivity is limited to the lowest excited state of a given spin multiplicity. Due to the close relationship with Kasha’s rule (which states that emission typically originates from the lowest excited state),^1,2^ the observed reactivity is nowadays often referred to as anti-Kasha reactivity.^5,32,56,57^ However, this terminology remains somewhat controversial, as Kasha’s rule was originally formulated to describe light emission, not chemical reactivity.^2^ Nevertheless, the kinetic parallels between emissive and reactive excited-state decay pathways justify the analogy,^9^ when reactivity arises from higher excited states within the same spin manifold. This distinction is important, as it differentiates the photoreactivity seen here from the more commonly observed reactivity involving S_1_ and T_1_ excited states.^7,58^

While previous synthetic-oriented research has speculated on solution based anti-Kasha reactivity,^31,32,55^ direct spectroscopic evidence has been lacking until now. Our findings highlight the critical role of preassociation in enabling static electron transfer on the picosecond time scale, countering the widespread belief that productive bimolecular photochemistry requires excited state lifetimes on the order of nanoseconds or longer. Such preassociation can be achieved through Coulombic interactions,^59–64^ π stacking,^5,32,65^ dipole–dipole interactions,^26,66^ or spatial confinement within supramolecular structures,^67^ leading to ultrafast electron transfer. However, our study also suggests that for anti-Kasha reactivity, the driving force must closely approach the reorganization energy (λ) associated with the photoinduced electron transfer, ensuring that the reaction is competitive with nonradiative energy dissipation. This requirement highlights the delicate balance between thermodynamics and kinetics in picosecond photochemistry. In the cases studied here, driving forces of 1.2 eV or more were required for anti-Kasha reactivity. Given that reorganization energies for electron transfer in artificial molecular systems are often between 0.8 and 1.2 eV,^45^ this insight provides a guiding principle for designing anti-Kasha reactivity far beyond the presently known examples.

The reductive dehalogenations used here were not aimed at establishing another synthetic method or tackling more challenging substrates.^68^ Instead, their purpose was to define the principles for achieving a fundamentally different reaction mode, distinct from more than 99% of the established photochemistry, and this key conceptual goal has been achieved. To date, anti-Kasha reactivity has been confined mainly to intramolecular processes such as isomerization reactions, photodissociation processes^69^ or electron transfer in covalently bound donor–acceptor compounds.^56^ Our study lays the groundwork for a broader use of anti-Kasha reactivity by providing design principles for bimolecular reactions.

The implications of anti-Kasha reactivity extend far beyond academic interest. By harnessing reactivity from short-lived, higher-energy states, catalytic systems can achieve unprecedented reactivities, with potential applications in selective pollutant degradation and chiral resolution.^26^ These findings expand the scope of photoredox catalysis and lay the foundation for exploring unconventional excited-state reactivity in organic and inorganic systems. Ultimately, this work redefines the energetic landscape of photocatalysis, providing new perspectives for leveraging higher excited states in synthetic methodologies and solar energy conversion.

## Supplementary Material



## Notes

General procedures and equipment details, photophysical and photochemical data (PDF)

## †

Department of Chemistry, Michigan State University, East Lansing, Michigan 48824, United States
