# Supplementary material for: Breaking Kasha’s Rule to Enable Higher Reactivity in Photoredox Catalysis
Source: J Am Chem Soc. 2025 Jul 17;147(30):26477–85. doi: 10.1021/jacs.5c06115 (PMC12314908; doi:10.1021/jacs.5c06115)
Supplement: Supplementary file 1 [file ja5c06115_si_001.pdf]

Supporting Information

Breaking Kasha's Rule to Enable Higher Reactivity in Photoredox Catalysis

Björn Pfund[a,#*], Oliver S. Wenger[[a]*]

[a] Department of Chemistry, University of Basel, St. Johanns-Ring 19, 4056 Basel, Switzerland

[#] Present Address: Department of Chemistry, Michigan State University, East Lansing, Michigan 48824, United States

Corresponding authors: bjoern.pfund@unibas.ch, oliver.wenger@unibas.ch

Materials and Methods

Optical spectroscopy

The solvents and reagents used for optical characterization and spectroscopic experiments were obtained commercially in high purity and were used without further purification. Synthetic procedures and characterization for 4,4''-dicyano-*p*-terphenyl (DCT) can be found in our previous report.[1]

Electrochemistry

Cyclic voltammetry (CV) measurements were conducted using a Versastat 3-200 potentiostat (Princeton Applied Research). The electrochemical setup consisted of a saturated calomel electrode (SCE) as the reference electrode, a glassy carbon disk as the working electrode, and a silver wire as the counter electrode. CV experiments were performed at a sweep rate of 50 mVs^{-1} in deaerated DMF solutions containing 0.1 M tetra-*n*-butylammonium hexafluorophosphate ($TBAPF_6$) as the supporting electrolyte and 1 mM analyte.

UV-Vis absorption spectro-electrochemistry was carried out in 1 mm optical path length cuvettes. The sample solution, containing 1 mM DCT and 0.1 M $TBAPF_6$ in deaerated DMF, was applied to a continuous voltage using a platinum mesh working electrode, a platinum wire counter electrode, and an Ag/AgCl reference electrode. The applied potential was controlled with the Versastat 3-200 potentiostat. UV-Vis absorption spectra were recorded using a Cary 5000 spectrophotometer (Varian) to monitor changes induced by the applied voltage.

Optical spectroscopy

Transient UV-Vis absorption spectroscopy in the microsecond time resolution was performed on an LP920-KS apparatus from Edinburgh Instruments. Excitation at 420 – 510 nm (10 – 14 mJ per pulse) was carried out by a frequency-tripled Nd:YAG pulsed laser (Quantel Brilliant, ca. 10 ns pulse width) equipped with an OPO from Opotek. For direct excitation of DCT at 355 nm, a frequency-tripled Nd:YAG laser (Quantel Brilliant b, ca. 10 ns pulse width, pulse energy <120 mJ) was used. The reactivity of the DCT radical anion was investigated using a frequency-doubled (532 nm) Nd:YAG pulsed laser (Quantel Brilliant b, ca. 10 ns pulse width, pulse energy <100 mJ) or direct (1064 nm) Nd:YAG pulsed laser (Quantel Brilliant b, ca. 10 ns pulse width, pulse energy <440 mJ). Synchronization of the two lasers and the detection system was achieved as described previously.[1] The excitation intensities of both lasers varied by the Q-switch delays and were measured with a COHERENT, Field MaxII-TOP Laser Power and Energy Meter. The beam diameter of 0.6 cm of the 355 nm lasers was enlarged using a beam expander (GBE02-A from

Thorlabs) to ensure homogeneous excitation of the 355 nm pulsed laser (beam diameter in front of the cuvette window was 1.3 cm) in the whole detection volume (about 1.2 cm^3). The 532 nm and 1064 nm beam diameters were not changed to obtain comparable light density. The lasers were carefully adjusted immediately before starting the measurements. The kinetic traces at a single wavelength were recorded using a photomultiplier tube.

<u>Nanosecond two-color pump-pump-probe experiment</u>

To ensure comparable conditions for excitation at 532 nm and 1064 nm, optical densities, photon counts, and laser pulse energies were carefully adjusted. Single-wavelength measurements were recorded at 500 nm using a photomultiplier tube. Given that the absorption coefficient of $DCT^{\bullet-}$ at 532 nm is ~2.5 times higher than at 1064 nm, the 355 nm laser pulse energy was tuned to equalize the transient absorption (TA) signal intensities at 532 and 1064 nm, respectively. Since direct TA detection at 1064 nm was not feasible, the resulting signal at 500 nm, is approximately 2.5-fold larger when using 1064 nm compared to 532 nm. Photon count was set to 3.6×10^{-7} Einstein per pulse, requiring the 1064 nm pulse to have half the energy of the 532 nm pulse.

The resulting signal intensities of ΔOD_{PP} (PP = pump-probe, changes in optical density immediately before the second pump pulse) and ΔOD_{PPP} (PPP = pump-pump-probe, change in optical density recorded after the second pump pulse) were averaged over a 75 ns interval. The grey shaded areas in the two-color pump–pump–probe plots indicate the corresponding average signal intensities (Figures S3 - S20). To avoid potential optical artifacts, the ΔOD_{PPP} values were extracted between 25 and 50 ns after the second laser pulse by averaging over a time interval of 75 ns, as marked by the second gray shaded zone in Figure 3a & b and Figures S6 - S20 of the main paper. This time window is short enough that changes in signal intensity solely dominated by the photoinduced ET, as the natural decay of $DCT^{\bullet-}$ occurs on a much longer timescale.

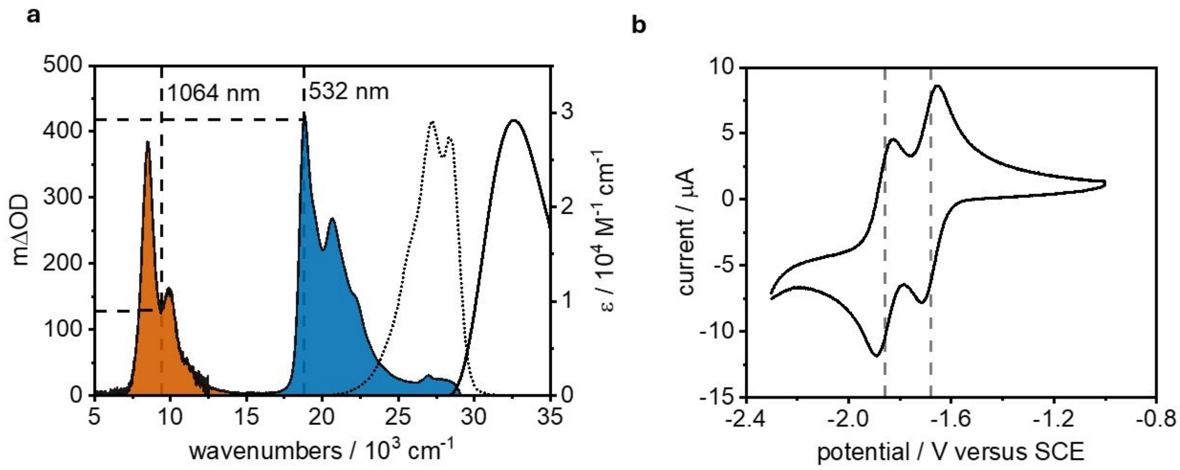

Figure S1. (a) UV-Vis absorption spectrum (solid black line), normalized luminescence spectra upon excitation at 320 nm (dotted black line) of DCT in DMF. Absorption changes following the electrochemical one-electron reduction of DCT at 20 °C in DMF are shown in the orange and blue shaded regions, representing the absorption bands for direct excitation into the D_1 and D_2 excited states of $DCT^{\bullet-}$, respectively. The dotted lines indicate the excitation wavelength of the two-color pump-pump-probe experiments at 532 nm and 1064 nm. (b) Cyclic voltammograms of argon-saturated DMF solutions containing 1 mM DCT and 0.1 M $TBAPF_6$ as a supporting electrolyte, using a saturated calomel reference electrode (SCE), a glassy carbon disk as a working electrode, and a silver wire as the counter electrode. The potential scan rate was 0.05 V s^{-1}.

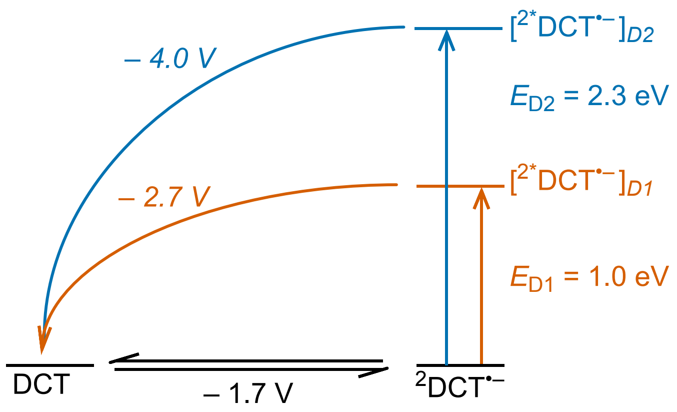

Figure S2. Latimer diagram derived from the electrochemical data in Figure 1b and the energies of the first (E_{D1}) and second (E_{D2}) doublet excited states. These energies were estimated from the low-energy side of the D_1 and D_2 absorption bands of DCT$^{\bullet-}$ (orange and blue shaded regions in Figure 1a), determined at the point where 10% of the maximum absorption is reached. The redox potentials are referenced to saturated calomel electrode (SCE).

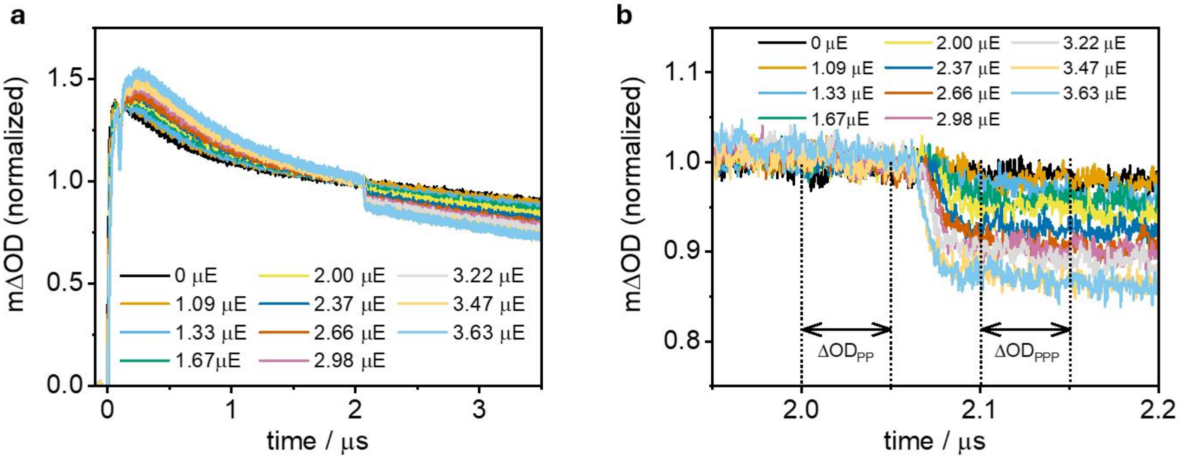

Figure S3. (a) Transient UV-Vis absorption kinetic decays of $DCT^{\bullet-}$ monitored at 500 nm using two-color pump-pump-probe spectroscopy. In this experiment, a 355 nm laser pulse (40 mJ) first generated $DCT^{\bullet-}$, followed by a second laser pulse up to 3.63 µE (408 mJ) after a time delay of ~2 µs, to excite $DCT^{\bullet-}$ at 1064 nm. These two-color pump-pump-probe experiments were performed using argon-saturated DMF solutions containing 2 mM DCT, 200 mM DMA, and 2 M chlorobenzene. All decay traces were normalized to a ΔOD_{PP} value of 1.0. (b) Magnified kinetic trace of the two-color pump-pump-probe experiments, where the 1064 nm pulse excited $DCT^{\bullet-}$. The arrows indicate the time windows, in which the signal intensities for ΔOD_{PP} (PP = pump-probe; change in optical density immediately before the second pump pulse) and ΔOD_{PPP} (PPP = pump-pump-probe, change in optical density recorded after the second pump pulse) were averaged (2.00 to 2.05 µs and 2.10 to 2.15 µs, respectively). To avoid potential optical artefacts, the ΔOD_{PPP} values were extracted by averaging the signal over a 50 ns time window starting approximately 25 ns after the second laser pulse.

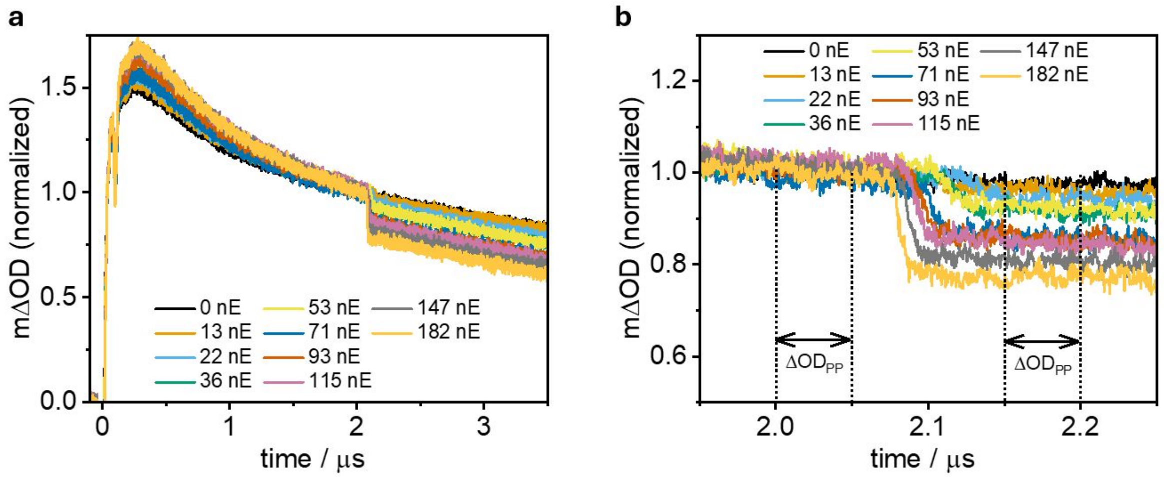

Figure S4. (a) Transient UV-Vis absorption kinetic decays of $DCT^{\bullet-}$ monitored at 500 nm using two-color pump-pump-probe spectroscopy. In this experiment, a 355 nm laser pulse (18 mJ) first generated $DCT^{\bullet-}$, followed by a second laser pulse up to 182 nE (41 mJ) after a time delay of ~2 μs, to excite $DCT^{\bullet-}$ at 532 nm. These two-color pump-pump-probe experiments were performed using argon-saturated DMF solutions containing 2 mM DCT, 200 mM DMA, and 2 M chlorobenzene. All decay traces were normalized to a ΔOD_{PP} (PP = pump-probe; change in optical density immediately before the second pump pulse) value of 1.0. (b) Magnified kinetic trace of the two-color pump-pump-probe experiments, where the 532 nm pulse excited $DCT^{\bullet-}$. The arrows indicate the time windows, in which the signal intensities for ΔOD_{PP} (PP = pump-probe; change in optical density immediately before the second pump pulse) and ΔOD_{PPP} (PPP = pump-pump-probe, change in optical density recorded after the second pump pulse) were averaged (2.00 to 2.05 μs and 2.15 to 2.20 μs, respectively). To avoid potential optical artefacts, the ΔOD_{PPP} values were extracted by averaging the signal over a 50 ns time window starting approximately 50 ns after the second laser pulse.

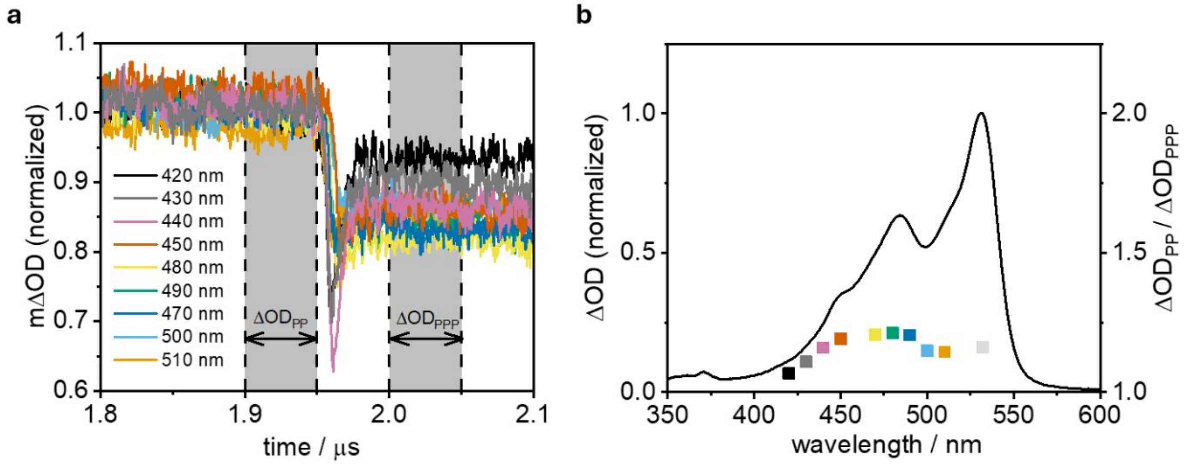

Figure S5. (a) Two-color pump-pump-probe experiment, where the 355 nm laser pulse (40 mJ) first generated $DCT^{•-}$, followed by a second laser excitation between 420 nm and 520 nm after a time delay of ~1.95 μs, to excite $DCT^{•-}$. To ensure the same amount of input energy, the laser pulses were set to a pulse power of 10 mJ, measured after sample holder. These two-color pump-pump-probe experiments were performed using argon-saturated DMF solutions containing 2 mM DCT, 200 mM DMA, and 2 M chlorobenzene. All decay traces were normalized to a ΔOD_{PP} value of 1.0. The arrows indicate the analyzed time windows for ΔOD_{PP} (PP = pump-probe, change in optical density immediately before the second pump pulse) at time delays from 1.90 μs to 1.95 μs and ΔOD_{PPP} (PPP = pump-pump-probe, change in optical density recorded after the second pump pulse) from 2.00 μs to 2.05 μs. To avoid potential optical artefacts, the ΔOD_{PPP} values were extracted by averaging the signal over a 50 ns time window starting approximately 50 ns after the second laser pulse. (b) Normalized absorption changes following the electrochemical one-electron reduction of DCT at 20 °C in DMF (black line). The data points represent a pseudo excitation spectrum, which matches the UV-Vis absorption spectrum of $DCT^{•-}$, at least in the accessible wavelength range.

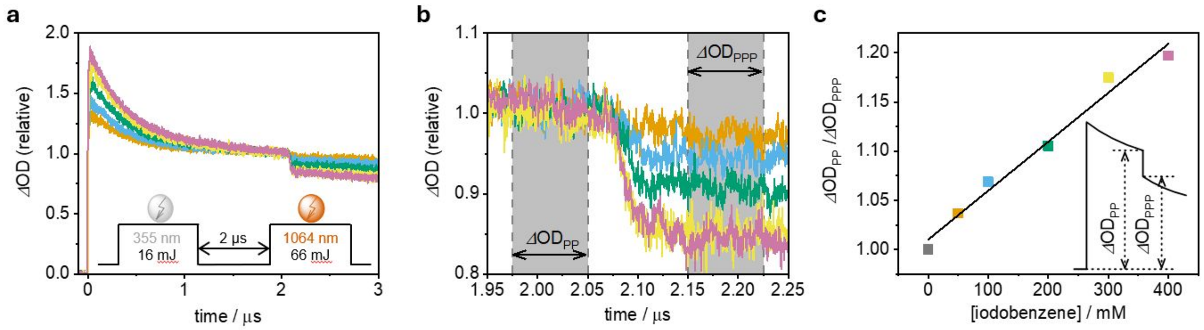

Figure S6. (a) Transient UV-Vis absorption kinetic decay of $DCT^{\bullet-}$ monitored at 500 nm in a two-color pump-pump-probe experiment, with the pulse scheme illustrated as an inset. In this experiment, a 355 nm laser pulse (16 mJ) first generates $DCT^{\bullet-}$. After a time delay of 2 μs, a second 1064 nm laser pulse (66 mJ) excites $DCT^{\bullet-}$. The experiment used an argon-saturated DMF solution containing 2 mM DCT, 200 mM DMA, and varying iodobenzene concentrations (0 mM and 400 mM). (b) Magnified kinetic decay trace of (a), showing the signal bleach caused by single electron transfer to iodobenzene following the second laser pulse. The grey shaded areas indicate the analyzed time windows for ΔOD_{PP} (PP = pump-probe, change in optical density immediately before the second pump pulse, from 1.975 μs to 2.050 μs) and ΔOD_{PPP} (PPP = pump-pump-probe, change in optical density recorded after the second pump pulse, from 2.150 μs to 2.225 μs). To avoid potential optical artefacts, the ΔOD_{PPP} values were extracted by averaging the signal over a 75 ns time window starting approximately 50 ns after the second laser pulse. (c) Stern-Volmer-like plot (identical to Figure 3d in the main manuscript) derived from the two-pulse experiment as a function of increasing iodobenzene concentration. Inset: Schematic representation of the two observables, ΔOD_{PP} and ΔOD_{PPP}. The values for ΔOD_{PP} and ΔOD_{PPP} were determined by averaging the signal intensity within the grey-shaded regions shown in (b).

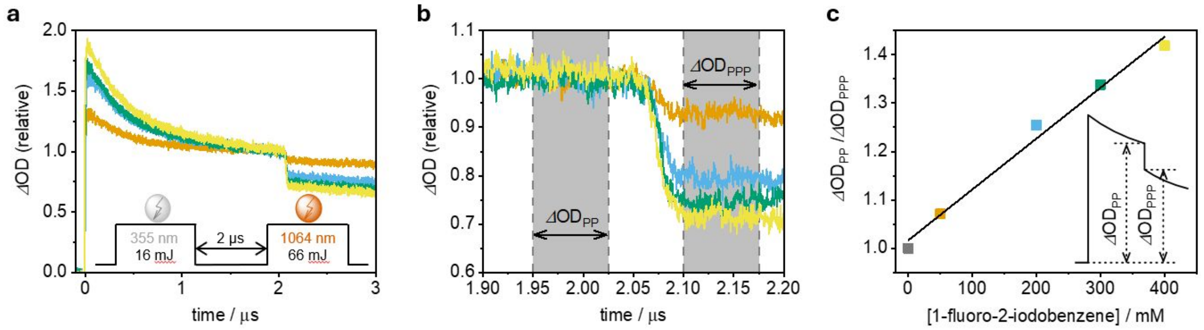

Figure S7. (a) Transient UV-Vis absorption kinetic decay of $DCT^{\bullet-}$ monitored at 500 nm in a two-color pump-pump-probe experiment, with the pulse scheme illustrated as an inset. In this experiment, a 355 nm laser pulse (16 mJ) first generates $DCT^{\bullet-}$. After a time delay of 2 µs, a second 1064 nm laser pulse (66 mJ) excites $DCT^{\bullet-}$. The experiment used an argon-saturated DMF solution containing 2 mM DCT, 200 mM DMA, and varying 1-fluoro-2-iodobenzene concentrations (0 mM and 400 mM). (b) Magnified kinetic decay trace of (a), showing the signal bleach caused by single electron transfer to 1-fluoro-2-iodobenzene following the second laser pulse. The grey shaded areas indicate the analyzed time windows for ΔOD_{PP} (PP = pump-probe, change in optical density immediately before the second pump pulse, from 1.950 µs to 2.025 µs) and ΔOD_{PPP} (PPP = pump-pump-probe, change in optical density recorded after the second pump pulse, from 2.100 µs to 2.175 µs). To avoid potential optical artefacts, the ΔOD_{PPP} values were extracted by averaging the signal over a 75 ns time window starting approximately 50 ns after the second laser pulse. (c) Stern-Volmer-like plot (identical to Figure 3d in the main manuscript) derived from the two-pulse experiment as a function of increasing 1-fluoro-2-iodobenzene concentration. Inset: Schematic representation of the two observables, ΔOD_{PP} and ΔOD_{PPP}. The values for ΔOD_{PP} and ΔOD_{PPP} were determined by averaging the signal intensity within the grey-shaded regions shown in (b).

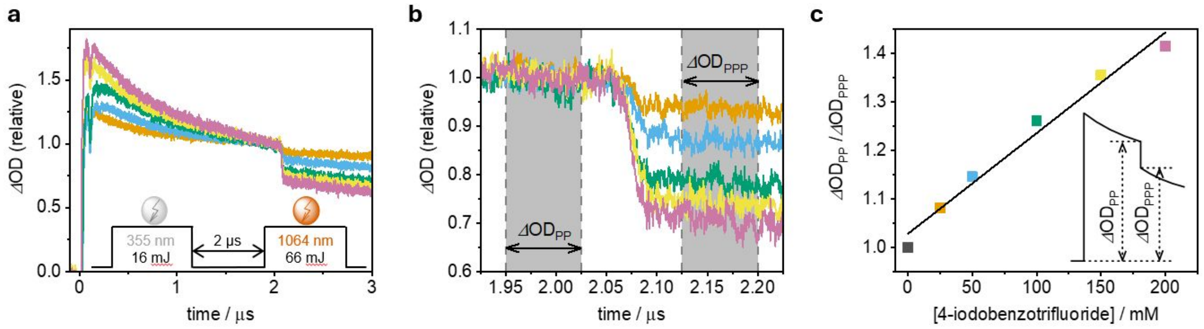

Figure S8. (a) Transient UV-Vis absorption kinetic decay of $DCT^{\bullet-}$ monitored at 500 nm in a two-color pump-pump-probe experiment, with the pulse scheme illustrated as an inset. In this experiment, a 355 nm laser pulse (16 mJ) first generates $DCT^{\bullet-}$. After a time delay of 2 μs, a second 1064 nm laser pulse (66 mJ) excites $DCT^{\bullet-}$. The experiment used an argon-saturated DMF solution containing 2 mM DCT, 200 mM DMA, and varying 4-iodobenzotrifluoride concentrations (0 mM and 200 mM). (b) Magnified kinetic decay trace of (a), showing the signal bleach caused by single electron transfer to 4-iodobenzotrifluoride following the second laser pulse. The grey shaded areas indicate the analyzed time windows for ΔOD_{PP} (PP = pump-probe, change in optical density immediately before the second pump pulse, from 1.950 μs to 2.025 μs) and ΔOD_{PPP} (PPP = pump-pump-probe, change in optical density recorded after the second pump pulse, from 2.125 μs to 2.200 μs). To avoid potential optical artefacts, the ΔOD_{PPP} values were extracted by averaging the signal over a 75 ns time window starting approximately 50 ns after the second laser pulse. (c) Stern-Volmer-like plot (identical to Figure 3d in the main manuscript) derived from the two-pulse experiment as a function of increasing 4-iodobenzotrifluoride concentration. Inset: Schematic representation of the two observables, ΔOD_{PP} and ΔOD_{PPP}. The values for ΔOD_{PP} and ΔOD_{PPP} were determined by averaging the signal intensity within the grey-shaded regions shown in (b).

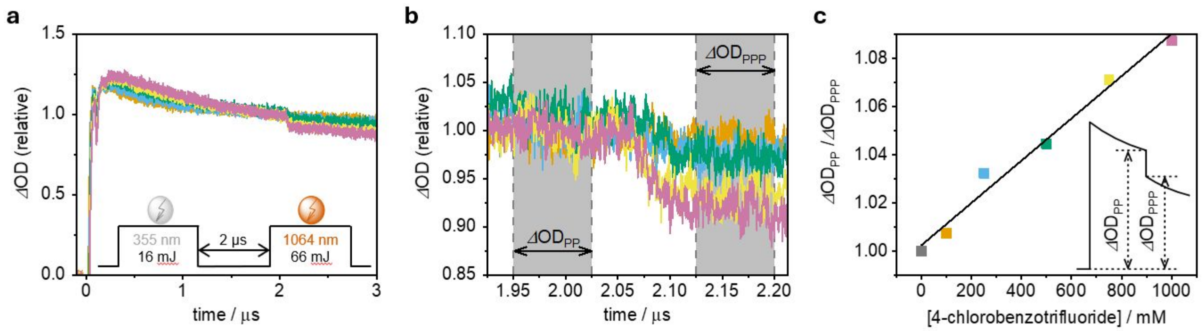

Figure S9. (a) Transient UV-Vis absorption kinetic decay of $DCT^{\bullet-}$ monitored at 500 nm in a two-color pump-pump-probe experiment, with the pulse scheme illustrated as an inset. In this experiment, a 355 nm laser pulse (16 mJ) first generates $DCT^{\bullet-}$. After a time delay of 2 μs, a second 1064 nm laser pulse (66 mJ) excites $DCT^{\bullet-}$. The experiment used an argon-saturated DMF solution containing 2 mM DCT, 200 mM DMA, and varying 4-chlorobenzotrifluoride concentrations (0 mM and 1000 mM). (b) Magnified kinetic decay trace of (a), showing the signal bleach caused by single electron transfer to 4-chlorobenzotrifluoride following the second laser pulse. The grey shaded time windows indicate the analyzed time windows for ΔOD_{PP} (PP = pump-probe, change in optical density immediately before the second pump pulse, from 1.950 μs to 2.025 μs) and ΔOD_{PPP} (PPP = pump-pump-probe, change in optical density recorded after the second pump pulse, from 2.125 μs to 2.200 μs). To avoid potential optical artefacts, the ΔOD_{PPP} values were extracted by averaging the signal over a 75 ns time window starting approximately 50 ns after the second laser pulse. (c) Stern-Volmer-like plot (identical to Figure 3d in the main manuscript) derived from the two-pulse experiment as a function of increasing 4-chlorobenzotrifluoride concentration. Inset: Schematic representation of the two observables, ΔOD_{PP} and ΔOD_{PPP}. The values for ΔOD_{PP} and ΔOD_{PPP} were determined by averaging the signal intensity within the grey-shaded regions shown in (b).

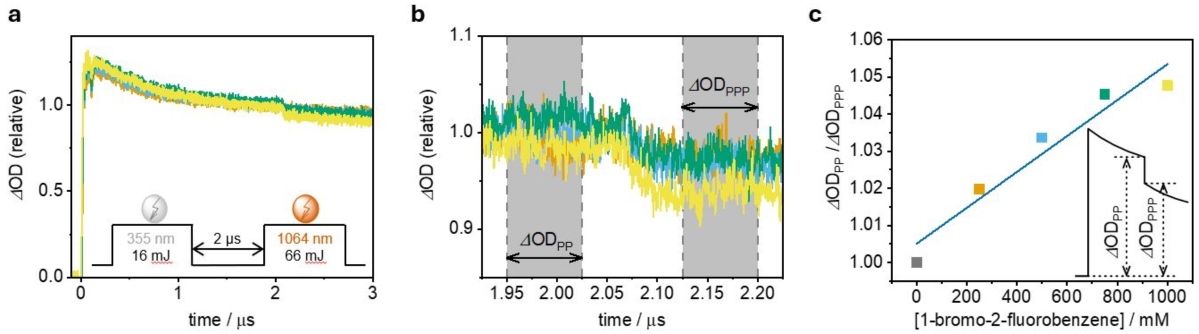

Figure S10. (a) Transient UV-Vis absorption kinetic decay of $DCT^{\bullet -}$ monitored at 500 nm in a two-color pump-pump-probe experiment, with the pulse scheme illustrated as an inset. In this experiment, a 355 nm laser pulse (16 mJ) first generates $DCT^{\bullet -}$. After a time delay of 2 µs, a second 1064 nm laser pulse (66 mJ) excites $DCT^{\bullet -}$. The experiment used an argon-saturated DMF solution containing 2 mM DCT, 200 mM DMA, and varying 1-bromo-2-fluorobenzene concentrations (0 mM and 1000 mM). (b) Magnified kinetic decay trace of (a), showing the signal bleach caused by single electron transfer to 1-bromo-2-fluorobenzene following the second laser pulse. The grey shaded areas indicate the analyzed time windows for ΔOD_{PP} (PP = pump-probe, change in optical density immediately before the second pump pulse, from 1.950 µs to 2.025 µs) and ΔOD_{PPP} (PPP = pump-pump-probe, change in optical density recorded after the second pump pulse, from 2.125 µs to 2.200 µs). To avoid potential optical artefacts, the ΔOD_{PPP} values were extracted by averaging the signal over a 75 ns time window starting approximately 50 ns after the second laser pulse. (c) Stern-Volmer-like plot (identical to Figure 3d in the main manuscript) derived from the two-pulse experiment as a function of increasing 1-bromo-2-fluorobenzene concentration. Inset: Schematic representation of the two observables, ΔOD_{PP} and ΔOD_{PPP}. The values for ΔOD_{PP} and ΔOD_{PPP} were determined by averaging the signal intensity within the grey-shaded regions shown in (b).

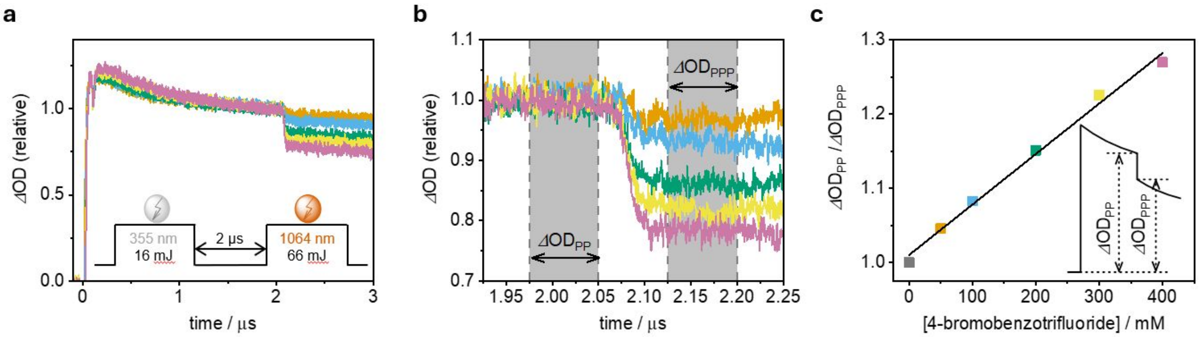

Figure S11. (a) Transient UV-Vis absorption kinetic decay of DCT$^{\bullet-}$ monitored at 500 nm in a two-color pump-pump-probe experiment, with the pulse scheme illustrated as an inset. In this experiment, a 355 nm laser pulse (16 mJ) first generates DCT$^{\bullet-}$. After a time delay of 2 µs, a second 1064 nm laser pulse (66 mJ) excites DCT$^{\bullet-}$. The experiment used an argon-saturated DMF solution containing 2 mM DCT, 200 mM DMA, and varying 4-bromobenzotrifluoride concentrations (0 mM and 400 mM). (b) Magnified kinetic decay trace of (a), showing the signal bleach caused by single electron transfer to 4-bromobenzotrifluoride following the second laser pulse. The grey shaded areas indicate the analyzed time windows for ΔOD_{PP} (PP = pump-probe, change in optical density immediately before the second pump pulse, from 1.975 µs to 2.050 µs) and ΔOD_{PPP} (PPP = pump-pump-probe, change in optical density recorded after the second pump pulse, from 2.125 µs to 2.200 µs). To avoid potential optical artefacts, the ΔOD_{PPP} values were extracted by averaging the signal over a 75 ns time window starting approximately 50 ns after the second laser pulse. (c) Stern-Volmer-like plot (identical to Figure 3d in the main manuscript) derived from the two-pulse experiment as a function of increasing 4-bromobenzotrifluoride concentration. Inset: Schematic representation of the two observables, ΔOD_{PP} and ΔOD_{PPP}. The values for ΔOD_{PP} and ΔOD_{PPP} were determined by averaging the signal intensity within the grey-shaded regions shown in (b).

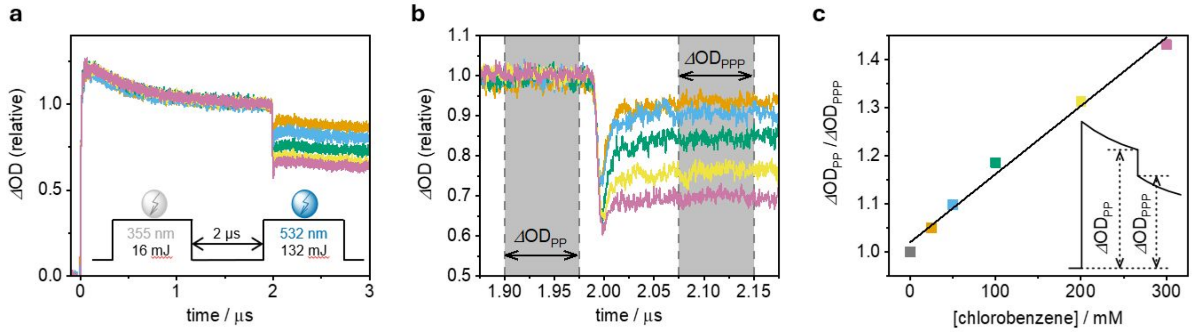

Figure S12. (a) Transient UV-Vis absorption kinetic decay of $DCT^{•-}$ monitored at 500 nm in a two-color pump-pump-probe experiment, with the pulse scheme illustrated as an inset. In this experiment, a 355 nm laser pulse (16 mJ) first generates $DCT^{•-}$. After a time delay of 2 μs, a second 532 nm laser pulse (132 mJ) excites $DCT^{•-}$. The experiment used an argon-saturated DMF solution containing 2 mM DCT, 200 mM DMA, and varying chlorobenzene concentrations (0 mM and 300 mM). (b) Magnified kinetic decay trace of (a), showing the signal bleach caused by single electron transfer to chlorobenzene following the second laser pulse. The grey shaded areas indicate the analyzed time windows for ΔOD_{PP} (PP = pump-probe, change in optical density immediately before the second pump pulse, from 1.950 μs to 2.025 μs) and ΔOD_{PPP} (PPP = pump-pump-probe, change in optical density recorded after the second pump pulse, from 2.075 μs to 2.150 μs). To avoid potential optical artefacts, the ΔOD_{PPP} values were extracted by averaging the signal over a 75 ns time window starting approximately 50 ns after the second laser pulse. (c) Stern-Volmer-like plot (identical to Figure 3c in the main manuscript) derived from the two-pulse experiment as a function of increasing chlorobenzene concentration. Inset: Schematic representation of the two observables, ΔOD_{PP} and ΔOD_{PPP}. The values for ΔOD_{PP} and ΔOD_{PPP} were determined by averaging the signal intensity within the grey-shaded regions shown in (b).

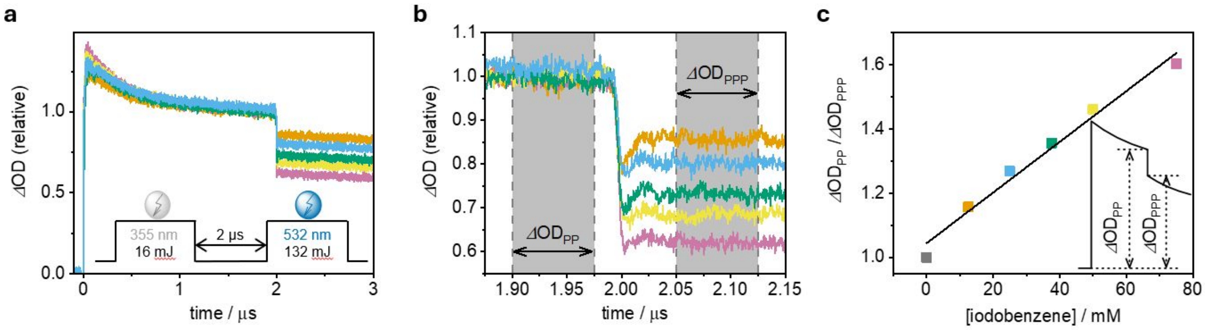

Figure S13. (a) Transient UV-Vis absorption kinetic decay of $DCT^{\bullet-}$ monitored at 500 nm in a two-color pump-pump-probe experiment, with the pulse scheme illustrated as an inset. In this experiment, a 355 nm laser pulse (16 mJ) first generates $DCT^{\bullet-}$. After a time delay of 2 μs, a second 532 nm laser pulse (132 mJ) excites $DCT^{\bullet-}$. The experiment used an argon-saturated DMF solution containing 2 mM DCT, 200 mM DMA, and varying iodobenzene concentrations (0 mM and 75 mM). (b) Magnified kinetic decay trace of (a), showing the signal bleach caused by single electron transfer to iodobenzene following the second laser pulse. The grey shaded areas indicate the analyzed time windows for ΔOD_{PP} (PP = pump-probe, change in optical density immediately before the second pump pulse, from 1.900 μs to 1.975 μs) and ΔOD_{PPP} (PPP = pump-pump-probe, change in optical density recorded after the second pump pulse, from 2.050 μs to 2.125 μs). To avoid potential optical artefacts, the ΔOD_{PPP} values were extracted by averaging the signal over a 75 ns time window starting approximately 50 ns after the second laser pulse. (c) Stern-Volmer-like plot (identical to Figure 3c in the main manuscript) derived from the two-pulse experiment as a function of increasing iodobenzene concentration. Inset: Schematic representation of the two observables, ΔOD_{PP} and ΔOD_{PPP}. The values for ΔOD_{PP} and ΔOD_{PPP} were determined by averaging the signal intensity within the grey-shaded regions shown in (b).

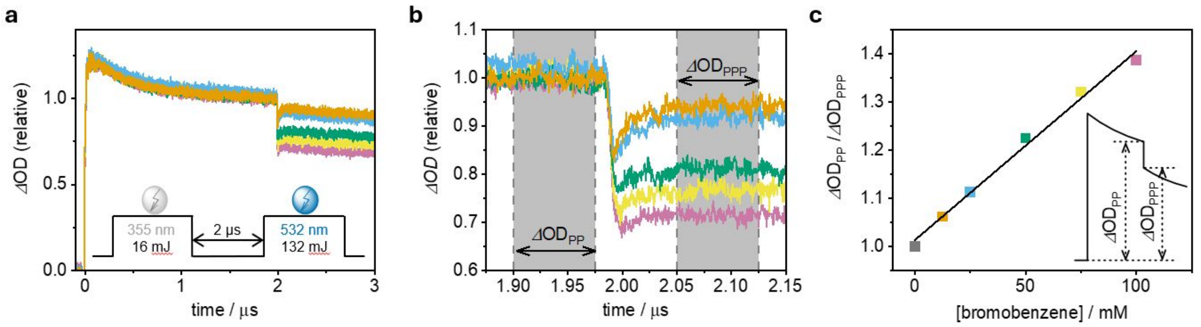

Figure S14. (a) Transient UV-Vis absorption kinetic decay of $DCT^{\bullet-}$ monitored at 500 nm in a two-color pump-pump-probe experiment, with the pulse scheme illustrated as an inset. In this experiment, a 355 nm laser pulse (16 mJ) first generates $DCT^{\bullet-}$. After a time delay of 2 μs, a second 532 nm laser pulse (132 mJ) excites $DCT^{\bullet-}$. The experiment used an argon-saturated DMF solution containing 2 mM DCT, 200 mM DMA, and varying bromobenzene concentrations (0 mM and 100 mM). (b) Magnified kinetic decay trace of (a), showing the signal bleach caused by single electron transfer to bromobenzene following the second laser pulse. The grey shaded areas indicate the analyzed time windows for ΔOD_{PP} (PP = pump-probe, change in optical density immediately before the second pump pulse, from 1.900 μs to 1.975 μs) and ΔOD_{PPP} (PPP = pump-pump-probe, change in optical density recorded after the second pump pulse, from 2.050 μs to 2.125 μs). To avoid potential optical artefacts, the ΔOD_{PPP} values were extracted by averaging the signal over a 75 ns time window starting approximately 50 ns after the second laser pulse. (c) Stern-Volmer-like plot (identical to Figure 3c in the main manuscript) derived from the two-pulse experiment as a function of increasing bromobenzene concentration. Inset: Schematic representation of the two observables, ΔOD_{PP} and ΔOD_{PPP}. The values for ΔOD_{PP} and ΔOD_{PPP} were determined by averaging the signal intensity within the grey-shaded regions shown in (b).

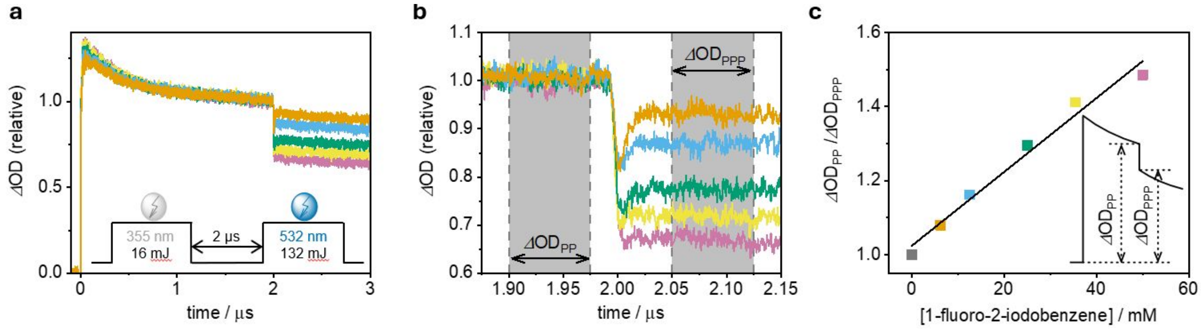

Figure S15*.* (a) Transient UV-Vis absorption kinetic decay of $DCT^{\bullet-}$ monitored at 500 nm in a two-color pump-pump-probe experiment, with the pulse scheme illustrated as an inset. In this experiment, a 355 nm laser pulse (16 mJ) first generates $DCT^{\bullet-}$. After a time delay of 2 μs, a second 532 nm laser pulse (132 mJ) excites $DCT^{\bullet-}$. The experiment used an argon-saturated DMF solution containing 2 mM DCT, 200 mM DMA, and varying 1-fluoro-2-iodobenzene concentrations (0 mM and 50 mM). (b) Magnified kinetic decay trace of (a), showing the signal bleach caused by single electron transfer to 1-fluoro-2-iodobenzene following the second laser pulse. The grey shaded areas indicate the analyzed time windows for ΔOD_{PP} (PP = pump-probe, change in optical density immediately before the second pump pulse, from 1.900 μs to 1.975 μs) and ΔOD_{PPP} (PPP = pump-pump-probe, change in optical density recorded after the second pump pulse, from 2.050 μs to 2.125 μs). To avoid potential optical artefacts, the ΔOD_{PPP} values were extracted by averaging the signal over a 75 ns time window starting approximately 50 ns after the second laser pulse. (c) Stern-Volmer-like plot (identical to Figure 3c in the main manuscript) derived from the two-pulse experiment as a function of increasing 1-fluoro-2-iodobenzene concentration. Inset: Schematic representation of the two observables, ΔOD_{PP} and ΔOD_{PPP}. The values for ΔOD_{PP} and ΔOD_{PPP} were determined by averaging the signal intensity within the grey-shaded regions shown in (b).

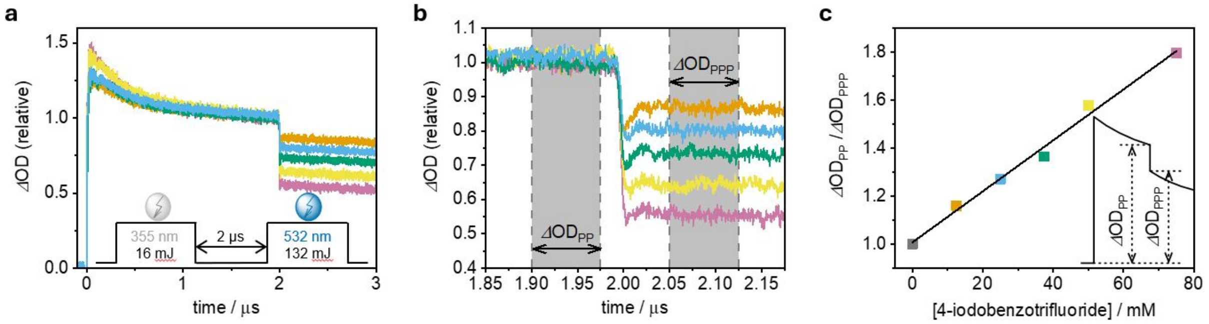

Figure S16. (a) Transient UV-Vis absorption kinetic decay of $DCT^{\bullet-}$ monitored at 500 nm in a two-color pump-pump-probe experiment, with the pulse scheme illustrated as an inset. In this experiment, a 355 nm laser pulse (16 mJ) first generates $DCT^{\bullet-}$. After a time delay of 2 μs, a second 532 nm laser pulse (132 mJ) excites $DCT^{\bullet-}$. The experiment used an argon-saturated DMF solution containing 2 mM DCT, 200 mM DMA, and varying 4-iodobenzotrifluoride concentrations (0 mM and 75 mM). (b) Magnified kinetic decay trace of (a), showing the signal bleach caused by single electron transfer to 4-iodobenzotrifluoride following the second laser pulse. The grey shaded areas indicate the analyzed time windows for ΔOD_{PP} (PP = pump-probe, change in optical density immediately before the second pump pulse, from 1.900 μs to 1.975 μs) and ΔOD_{PPP} (PPP = pump-pump-probe, change in optical density recorded after the second pump pulse, from 2.050 μs to 2.125 μs). To avoid potential optical artefacts, the ΔOD_{PPP} values were extracted by averaging the signal over a 75 ns time window starting approximately 50 ns after the second laser pulse. (c) Stern-Volmer-like plot (identical to Figure 3c in the main manuscript) derived from the two-pulse experiment as a function of increasing 4-iodobenzotrifluoride concentration. Inset: Schematic representation of the two observables, ΔOD_{PP} and ΔOD_{PPP}. The values for ΔOD_{PP} and ΔOD_{PPP} were determined by averaging the signal intensity within the grey-shaded regions shown in (b).

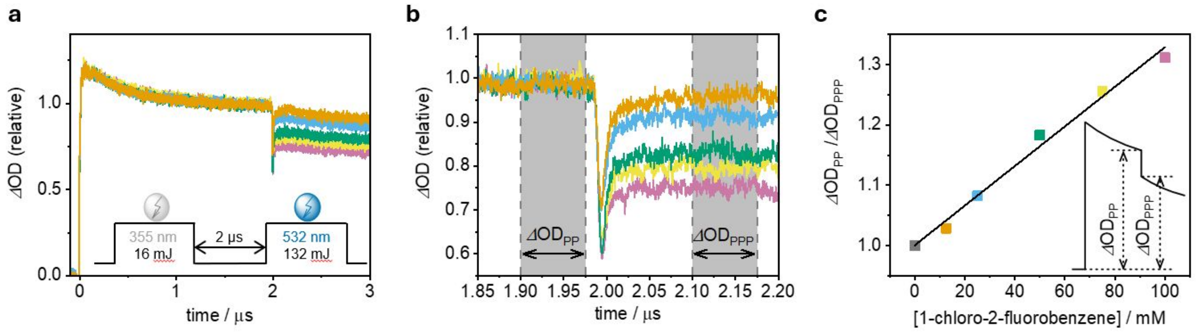

Figure S17. (a) Transient UV-Vis absorption kinetic decay of $DCT^{\bullet-}$ monitored at 500 nm in a two-color pump-pump-probe experiment, with the pulse scheme illustrated as an inset. In this experiment, a 355 nm laser pulse (16 mJ) first generates $DCT^{\bullet-}$. After a time delay of 2 μs, a second 532 nm laser pulse (132 mJ) excites $DCT^{\bullet-}$. The experiment used an argon-saturated DMF solution containing 2 mM DCT, 200 mM DMA, and varying 1-chloro-2-fluorobenzene concentrations (0 mM and 100 mM). (b) Magnified kinetic decay trace of (a), showing the signal bleach caused by single electron transfer to 1-chloro-2-fluorobenzene following the second laser pulse. The grey shaded areas indicate the analyzed time windows for ΔOD_{PP} (PP = pump-probe, change in optical density immediately before the second pump pulse, from 1.900 μs to 1.975 μs) and ΔOD_{PPP} (PPP = pump-pump-probe, change in optical density recorded after the second pump pulse, from 2.100 μs to 2.175 μs). To avoid potential optical artefacts, the ΔOD_{PPP} values were extracted by averaging the signal over a 75 ns time window starting approximately 50 ns after the second laser pulse. (c) Stern-Volmer-like plot (identical to Figure 3c in the main manuscript) derived from the two-pulse experiment as a function of increasing 1-chloro-2-fluorobenzene concentration. Inset: Schematic representation of the two observables, ΔOD_{PP} and ΔOD_{PPP}. The values for ΔOD_{PP} and ΔOD_{PPP} were determined by averaging the signal intensity within the grey-shaded regions shown in (b).

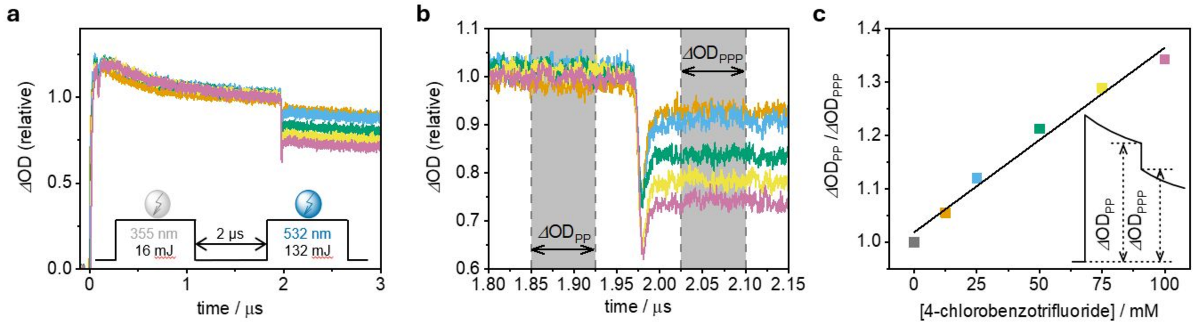

Figure S18. (a) Transient UV-Vis absorption kinetic decay of $DCT^{\bullet-}$ monitored at 500 nm in a two-color pump-pump-probe experiment, with the pulse scheme illustrated as an inset. In this experiment, a 355 nm laser pulse (16 mJ) first generates $DCT^{\bullet-}$. After a time delay of 2 µs, a second 532 nm laser pulse (132 mJ) excites $DCT^{\bullet-}$. The experiment used an argon-saturated DMF solution containing 2 mM DCT, 200 mM DMA, and varying 4-chlorobenzotrifluoride concentrations (0 mM and 100 mM). (b) Magnified kinetic decay trace of (a), showing the signal bleach caused by single electron transfer to 4-chlorobenzotrifluoride following the second laser pulse. The grey shaded areas indicate the analyzed time windows for ΔOD_{PP} (PP = pump-probe, change in optical density immediately before the second pump pulse, from 1.850 µs to 1.925 µs) and ΔOD_{PPP} (PPP = pump-pump-probe, change in optical density recorded after the second pump pulse, from 2.025 µs to 2.100 µs). To avoid potential optical artefacts, the ΔOD_{PPP} values were extracted by averaging the signal over a 75 ns time window starting approximately 50 ns after the second laser pulse. (c) Stern-Volmer-like plot (identical to Figure 3c in the main manuscript) derived from the two-pulse experiment as a function of increasing 4-chlorobenzotrifluoride concentration. Inset: Schematic representation of the two observables, ΔOD_{PP} and ΔOD_{PPP}. The values for ΔOD_{PP} and ΔOD_{PPP} were determined by averaging the signal intensity within the grey-shaded regions shown in (b).

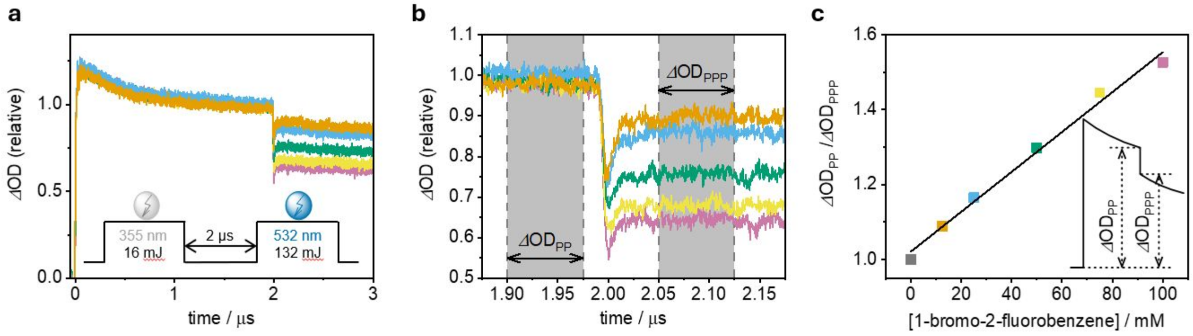

Figure S19. (a) Transient UV-Vis absorption kinetic decay of $DCT^{•–}$ monitored at 500 nm in a two-color pump-pump-probe experiment, with the pulse scheme illustrated as an inset. In this experiment, a 355 nm laser pulse (16 mJ) first generates $DCT^{•–}$. After a time delay of 2 μs, a second 532 nm laser pulse (132 mJ) excites $DCT^{•–}$. The experiment used an argon-saturated DMF solution containing 2 mM DCT, 200 mM DMA, and varying 1-bromo-2-fluorobenzene concentrations (0 mM and 100 mM). (b) Magnified kinetic decay trace of (a), showing the signal bleach caused by single electron transfer to 1-bromo-2-fluorobenzene following the second laser pulse. The grey shaded areas indicate the analyzed time windows for ΔOD_{PP} (PP = pump-probe, change in optical density immediately before the second pump pulse, from 1.900 μs to 1.975 μs) and ΔOD_{PPP} (PPP = pump-pump-probe, change in optical density recorded after the second pump pulse, from 2.050 μs to 2.125 μs). To avoid potential optical artefacts, the ΔOD_{PPP} values were extracted by averaging the signal over a 75 ns time window starting approximately 50 ns after the second laser pulse. (c) Stern-Volmer-like plot (identical to Figure 3c in the main manuscript) derived from the two-pulse experiment as a function of increasing 1-bromo-2-fluorobenzene concentration. Inset: Schematic representation of the two observables, ΔOD_{PP} and ΔOD_{PPP}. The values for ΔOD_{PP} and ΔOD_{PPP} were determined by averaging the signal intensity within the grey-shaded regions shown in (b).

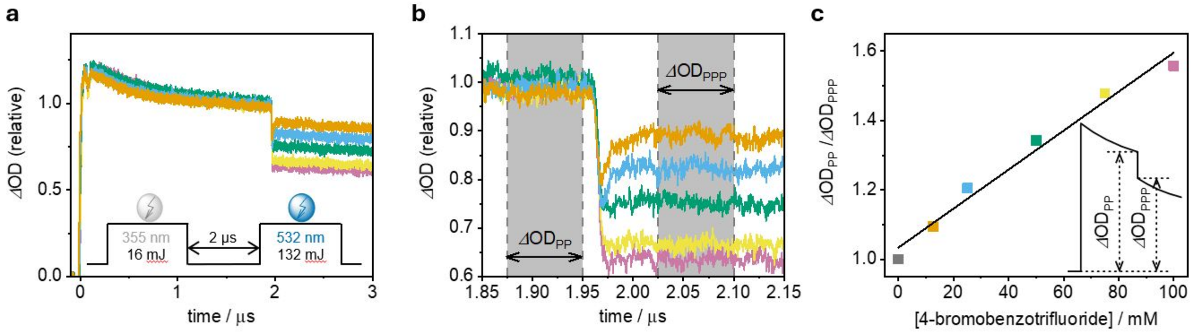

Figure S20. (a) Transient UV-Vis absorption kinetic decay of $DCT^{\bullet-}$ monitored at 500 nm in a two-color pump-pump-probe experiment, with the pulse scheme illustrated as an inset. In this experiment, a 355 nm laser pulse (16 mJ) first generates $DCT^{\bullet-}$. After a time delay of 2 µs, a second 532 nm laser pulse (132 mJ) excites $DCT^{\bullet-}$. The experiment used an argon-saturated DMF solution containing 2 mM DCT, 200 mM DMA, and varying 4-bromobenzotrifluoride concentrations (0 mM and 100 mM). (b) Magnified kinetic decay trace of (a), showing the signal bleach caused by single electron transfer to 4-bromobenzotrifluoride following the second laser pulse. The grey shaded areas indicate the analyzed time windows for ΔOD_{PP} (PP = pump-probe, change in optical density immediately before the second pump pulse, from 1.875 µs to 1.950 µs) and ΔOD_{PPP} (PPP = pump-pump-probe, change in optical density recorded after the second pump pulse, from 2.025 µs to 2.100 µs). To avoid potential optical artefacts, the ΔOD_{PPP} values were extracted by averaging the signal over a 75 ns time window starting approximately 50 ns after the second laser pulse. (c) Stern-Volmer-like plot (identical to Figure 3c in the main manuscript) derived from the two-pulse experiment as a function of increasing 4-bromobenzotrifluoride concentration. Inset: Schematic representation of the two observables, ΔOD_{PP} and ΔOD_{PPP}. The values for ΔOD_{PP} and ΔOD_{PPP} were determined by averaging the signal intensity within the grey-shaded regions shown in (b).

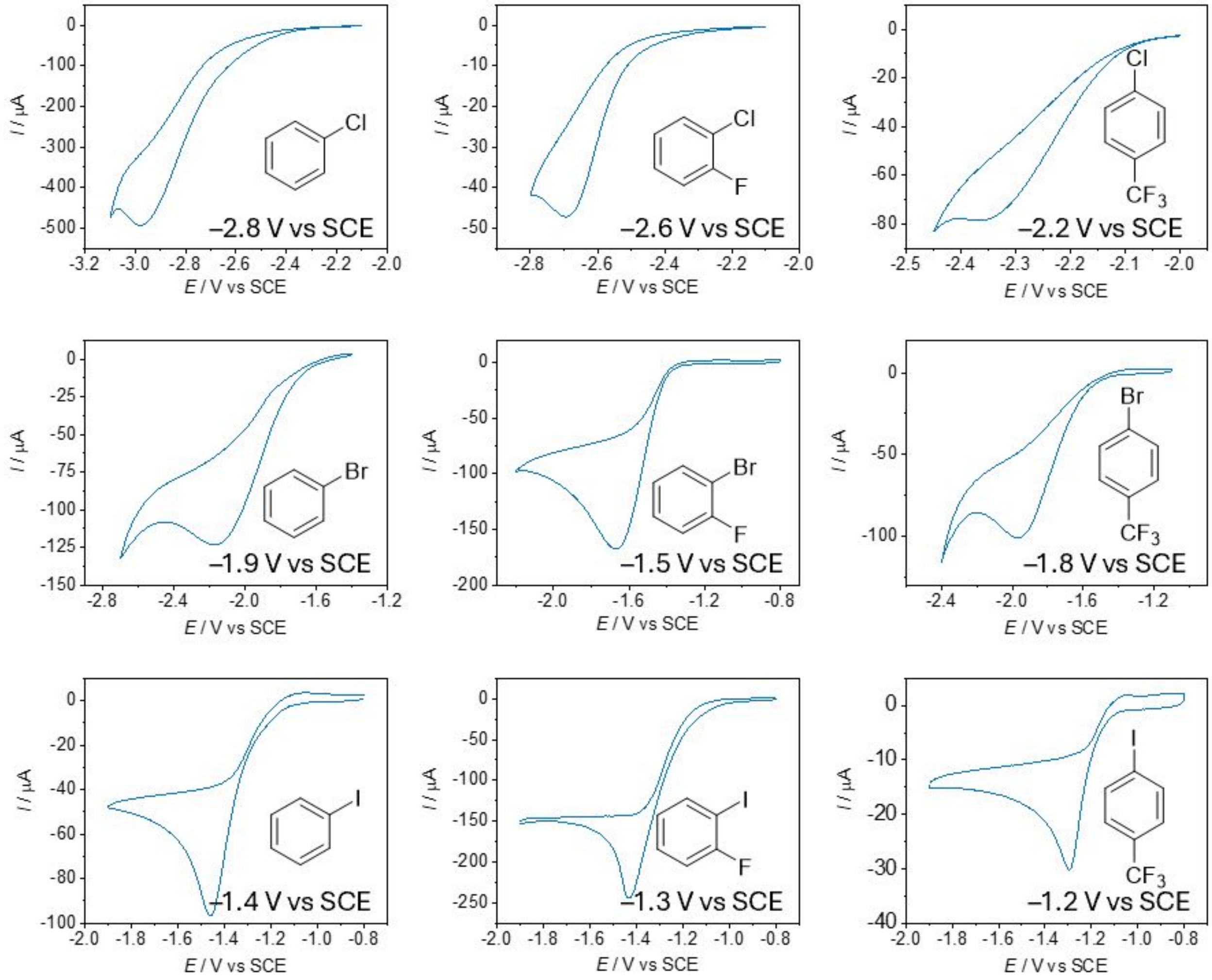

Figure S21. Cyclic voltammograms of electron acceptors measured with $TBAPF_6$ (0.1 M) as supporting electrolyte in deaerated DMF. Voltammograms were recorded with scan rates of 0.05 V s^{-1}.

Table S1. Summary of the electron acceptors' redox potential, driving forces for photoinduced electron transfer from the D_1 or D_2 states, and the pseudo Stern-Volmer constants obtained after excitation at different wavelengths.

	E_{red} / V versus SCE [a]	$\Delta G_{ET(D1)}^0$/ V [b]	K_{SV} (1064 nm) / M^{-1} [c]	$\Delta G_{ET(D2)}^0$/ V [d]	K_{SV} (532 nm) / M^{-1} [e]
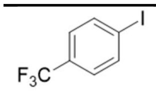	−1.2	−1.5	2.07	−2.8	10.6
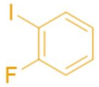	−1.3	−1.4	1.05	−2.7	9.98
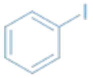	−1.4	−1.3	0.50	−2.6	7.89
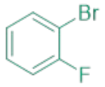	−1.5	−1.2	0.05	−2.5	5.33
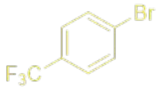	−1.8	−0.9	0.68	−2.2	5.62
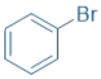	−1.9	−0.8	n/d	−2.1	3.93
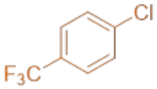	−2.2	−0.5	0.09	−1.8	3.45
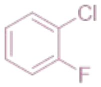	−2.6	−0.1	n/d	−1.4	3.26
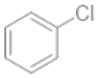	−2.8	+0.1	n/d	−1.2	1.42
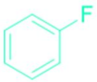	−3.0[1]	+0.3	n/d	−1.0	n/d

[a] Redox potentials were determined using cyclic voltammetry (see Figure S21 for details). [b] The driving force was calculated considering the maximum excited-state redox potential of −2.7 V versus SCE, corresponding to the lowest excited state of $DCT^{\bullet-}$ and the reduction potentials of the electron donors (column 2). [c] Pseudo Stern-Volmer constant of the lowest excited state, determined by two-color, two-pulse laser flash photolysis, using 1064 nm as the second laser pulse (for details, see Figures S6–11). [d] The driving force was calculated considering the maximum excited-state redox potential of −4.0 V versus SCE, corresponding to the second excited state of $DCT^{\bullet-}$ and the reduction potentials of the electron donors (column 2). [e] Pseudo Stern-Volmer constant of the higher excited state, determined by two-color, two-pulse laser flash photolysis, using 532 nm as the second laser pulse (for details, see Figures S12–20).

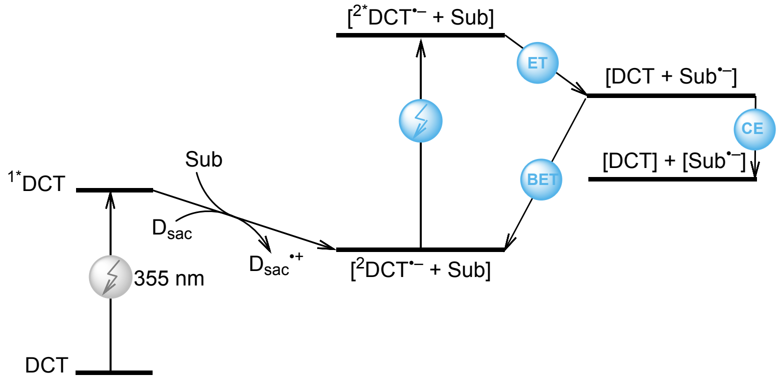

Figure S22. Cage escape effect in two-color pump–pump–probe experiments. Excitation of DCT at 355 nm in the presence of a sacrificial electron donor (D_{sac}) generates DCT$^{•−}$. In the presence of a substrate (Sub), pre-association can occur. Subsequent selective excitation of DCT$^{•−}$ into one of its doublet excited states using 1064 or 532 nm light enables productive electron transfer (ET) to the substrate, forming an in-cage photoproduct ([DCT + Sub$^{•−}$]). If back electron transfer (BET) is faster than cage escape (CE), no signal bleach of DCT$^{•−}$ is observed, as the overall absorption features remain unchanged (before and after the second pulse excitation). Thus, signal bleach is only detected after successful cage escape.

References

(1) Pfund, B.; Gejsnæs-Schaad, D.; Lazarevski, B.; Wenger, O. S. Monitoring picosecond reactions of excited radical ion super reductants in photocatalysis. *Nat. Commun.* **2024**, *15*, 4738. DOI: 10.1038/s41467-024-49006-5.
